# A fast and frugal Gaussian boson sampling emulator

**DOI:** 10.1126/sciadv.aef8431

**Published:** 2026-10-14

**Authors:** Tom Dodd, Javier Martínez-Cifuentes, Oliver Thomson Brown, Nicolás Quesada, Raúl García-Patrón

**Affiliations:** ^1^ School of Informatics, University of Edinburgh, 10 Crichton Street, Edinburgh EH8 9AB, UK.; ^2^ Département de génie physique, École polytechnique de Montréal, Montréal, QC H3T 1J4, Canada.; ^3^ EPCC, University of Edinburgh, 47 Potterow, Edinburgh EH8 9BT, UK.; ^4^ Phasecraft Ltd., 77 Charlotte Street, London W1T 4PW, UK.

## Abstract

Classical algorithms have successfully reproduced expectation values of observables for certain quantum circuits using off-the-shelf computing resources; however, matching the performance of the most advanced quantum devices on sampling problems typically incurs extreme memory and computational costs, restricting such simulations to only a handful of supercomputers worldwide. In this work, we demonstrate a classical simulation outperforming Gaussian boson sampling experiments of 100 modes on established benchmark tests using a single central processing unit (CPU) or graphics processing unit (GPU). Being embarrassingly parallelizable, a small number of CPUs or GPUs allows us to match previous sampling rates that required more than 100 GPUs. We believe that algorithmic and implementation improvements have the potential to generalize our tools to photocounting and pseudo–photon number–resolving scenarios and larger system sizes. Last, most of the innovations in our tools remain valid for generic probability distributions over binary variables, rendering them potentially applicable to the simulation of qubit-based sampling problems and creating classical surrogates for classical-quantum algorithms.

## INTRODUCTION

The past 6 years have seen an increasing number of implementations of quantum algorithms claiming to have reached quantum computational advantage over their classical counterparts; these range from random circuit sampling on quantum superconducting devices (*1*, *2*) and Gaussian or single-photon boson sampling on photonic hardware (*3*–*7*) and atomic ensembles (*8*), respectively, to the estimation of dynamical observables in trapped ions (*9*) and superconducting circuits (*10*). On hypothetical error-free quantum hardware, all the candidate proposals are believed to be out of reach for classical computers, challenging today’s most advanced supercomputers with sizes beyond 50 qubits or hundreds of modes and photons. For sampling problems, the hardness of their classical simulation is often backed by complexity-theoretic arguments (*11*–*15*).

In reality, current quantum devices are prone to errors and imperfections that limit their performance. Unless fault tolerance based on quantum error correction is implemented, the quality of the computations decays rapidly. The pervasive presence of errors is a key enabler behind classical algorithm implementations that claim to have successfully disproved previous claims of quantum advantage while remaining as faithful as possible to the system’s true behavior rather than spoofing the quantum certification test. Some examples are refs. (*16*, *17*) for random quantum circuits, ref. (*18*) for Gaussian boson sampling (GBS), and refs. (*19*–*21*) for the estimation of observables of complex spin system dynamics.

While classical algorithms have been successful in reproducing the estimation of expectation values of observables of some recent quantum experiments, even using off-the-shelf computing resources (*19*–*21*), the simulation of sampling tasks, both qubit- and bosonic-based, is known to pose substantially harder challenges to classical simulations. The implementations of these algorithms required an extreme cost in terms of memory and computing operations, making them accessible to only a handful of supercomputers around the world and unreasonable to reproduce on a regular basis. To date, there has been no demonstration of a classical algorithm that matches the performance on standard benchmarking tests of the most advanced quantum devices on a sampling problem using off-the-shelf computational resources.

In this work, we demonstrate a classical simulation of imperfect GBS matching and sometimes outperforming the actual quantum experiments in a series of standardized tests, requiring only a single central processing unit (CPU) or graphics processing unit (GPU), while ref. (*18*) required more than 100 GPUs to perform essentially equivalently to our sampler (see comparison in section S5). In addition, our implementation for 144 modes requires 2 gigabytes to encode the quantum state before the measurement, three orders of magnitude less than in (*18*). Our algorithm does not require gradient-descent training like Boltzmann machine–based emulators or suffer from the strongly underdispersed samples of the Greedy approach, both in (*22*). Being embarrassingly parallelizable, we were able to generate 10 million samples in 115 min using eight contemporary GPUs on one single cluster node. These notable improvements make mainstream what was before accessible only to a handful of facilities in the world.

Our main technical contribution is the design of an algorithm to sample from the joint probabilities of *M* bit samples by exploiting decomposition in terms of cumulants of increasing order and approximated marginals of the original distribution. Truncating the expression to a constant order *K* allows us to capture all relevant statistics of the hardware experiment while making its running timescale as $O\left(M^{K}\right)$.

Because the algorithm benefits from polynomial scaling of order *K*, it has the potential to compete against future hardware improvements and larger system sizes. We believe that the algorithm is easily generalizable to photocounting. Last, most of the innovations in our tools remain valid for generic probability distributions over binary variables, rendering them applicable to the simulation of qubit-based sampling problems and creating classical surrogates for hybrid classical-quantum algorithms.

## RESULTS

### Emulator description

Error-free GBS is believed to be a hard sampling problem where an instance over *M* modes is defined by a set of squeezed state sources located at a given set of input ports and a linear-optical interferometer, characterized by an M×M unitary matrix *U* selected randomly over the Haar measure. Except for the Borealis experiment (*5*), where photocounting was implemented at the output, all other GBS demonstrations so far have implemented threshold detection (*3*, *4*, *6*). In this work, we focus on the second type of GBS, consisting of binary outcomes encoding the absence or presence of photons.

Historically, the ground truth of the GBS experiment used in its validation tests is the description of the Gaussian state at the output of the real interferometer taking into account the presence of losses. This approach is different from the one used in, for example, random circuit sampling, where one computes benchmarks such as the cross linear entropy with respect to the ideal noiseless instance. Whether it is more appropriate to use a lossy ground truth for photonic quantum advantage remains an open research problem (*23*) [and also the very recent preprint (*24*)]. With our goal being able to design an emulator of noisy GBS, it is natural and noncontroversial for us to consider the lossy ground truth as the benchmark to compare.

As described in Fig. 1, the ground truth is obtained from a characterization of the GBS device’s transmission matrix and squeezing parameters. Alternatively, one could envision constructing the ground truth from the ideal GBS instance combined with an accurate model of imperfections. Figure 1 summarizes our emulator as a two-step process. We start from the ground truth Gaussian state’s (2M×2M) covariance matrix and compute $O\left(M^{K}\right)$ Fourier coefficients up to order *K* and the corresponding cumulants, following the methodology explained in the “Precomputing the cumulants” section. The choice of the truncation order *K* was made heuristically from a study of the experimental data, although one could develop and exploit analytical tools bounding the quality of the approximation as a function of *K*. The algorithm’s handling of the cumulants requires a data structure preprocessing task that only needs to be run once for each choice of *M* and *K*, irrespective of the interferometric circuit. As we detail later in the “Cost estimation and scaling” section, numerics hint to $O\left(M^{K+1.5}\right)$ scaling of this data structure preprocessing.

**Fig. 1.**
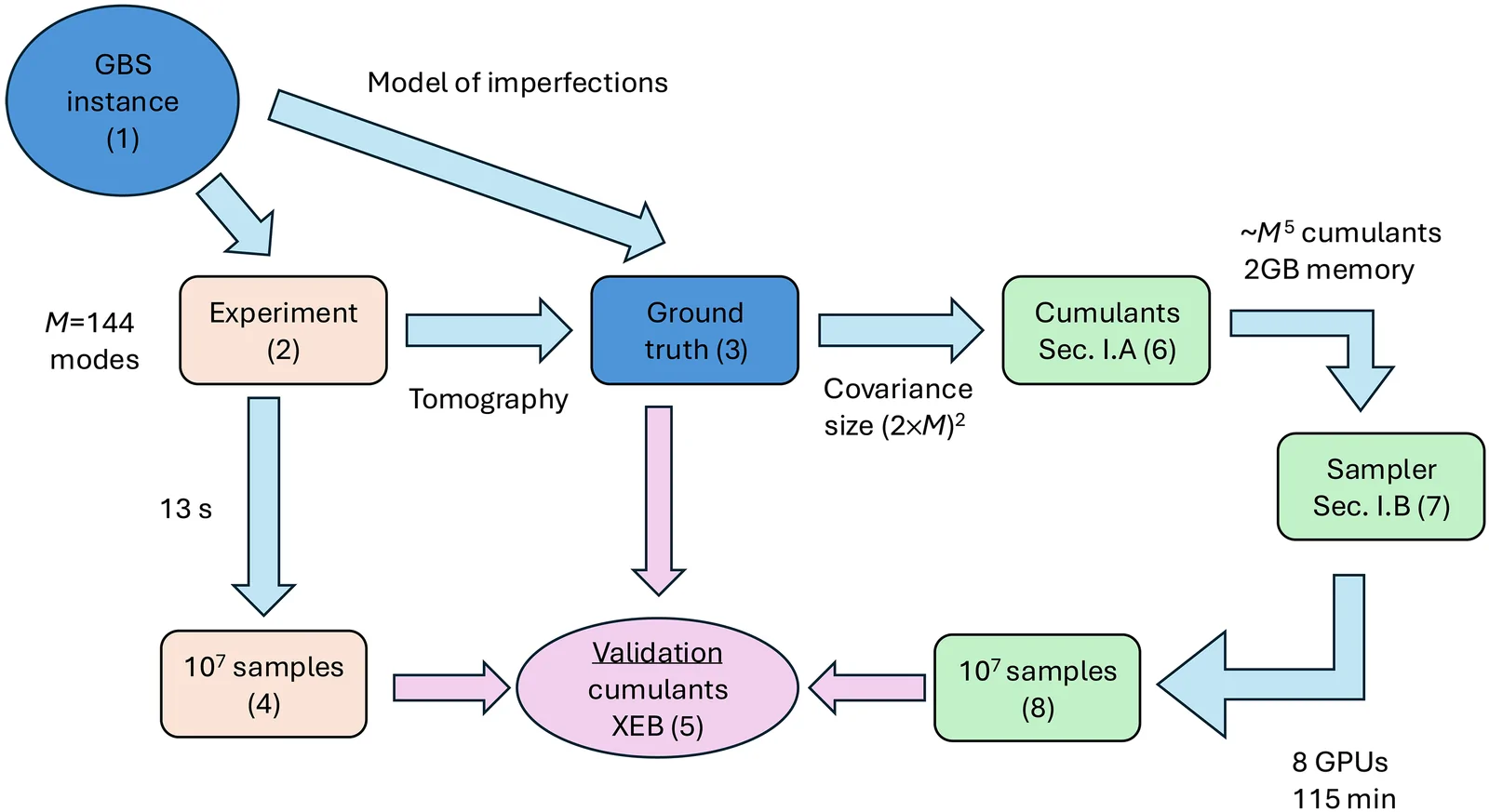
Simulation and validation workflow. (1) An instance of GBS is characterized by its ideal optical circuit or, equivalently, the covariance matrix of its associated Gaussian state. (2) An experiment implements a version affected by losses and other imperfections. (3) One can extract the ground truth of the experiment from tomographic data or apply a good model of imperfections to the noiseless GBS instance. (4) Ten million samples are generated by quantum hardware in ∼10 s. (5) The samples are benchmarked using cumulant statistical analysis and XEB. (6) The first stage of our algorithm computes a list of $\left(\begin{array}{c} 144\\ 5 \end{array}\right)$ correlators that are later transformed into cumulants from the ground truth covariance matrix, as detailed in the “Precomputing the cumulants” section. (7) The second stage generates samples from the corresponding cumulants as detailed in the “Sampling algorithm” section. (8) Using eight GPUs, we can generate 10 million samples in 115 min, later validated with the same benchmarking tests.

The second step is a sampling algorithm described in the “Sampling algorithm” section, which generates samples using a chain rule of conditional probabilities, where each new bit is chosen according to the bias between 0 and 1 that is computed from a large weighted sum composed of precomputed cumulants and approximations of marginals, which are computed following a dynamical programming approach.

The certification of large-scale GBS is often performed using a number of different metrics. First, Bayesian and heavy output generation tests have been used to compare the experimental data against different hypotheses or spoofers/simulators, respectively. More robust approaches are the comparison of the click cumulants and a variant of cross-entropy benchmarking (XEB) that focuses on a postselected set of data within a range of total photon counts, which have been used to provide evidence of quantum advantage by hardware implementations (*3*–*6*). Therefore, we will follow the recent classical algorithm in (*18*) and use cross-linear entropy and cumulant distributions to benchmark our emulator.

#### Precomputing the cumulants

A cumulant κ(S) of order *d* captures all “novel” correlations present in a *d*-mode marginal distribution that are not depicted by its lower-order cumulants of orders 1 through *d* − 1. This property is nicely characterized by the recursive definition of a cumulant for a set of random variables $S=\left\{i_{1},\ldots ,i_{d}\right\}$, where $i_{j}\in \left[M\right]\equiv \left\{1,\ldots ,M\right\}$

$$\kappa \left(S\right)=c\left(S\right)-\sum_{\pi \in P\left(S\right)\backslash \left\{S\right\}}\prod_{B\in \pi }\kappa \left(B\right)$$ (1)

where P(S) is the set of partitions of *S*, $\chi \left(A\right)={\left(-1\right)}^{\sum_{k\in A}x_{k}}$ is the parity function over the set of variables *A*, and $c\left(A\right)=\sum_{{\bar{x}}_{A}\in {\left\{0,1\right\}}^{|A|}}\chi_{A}\left(\bar{x}\right)\;p\left({\bar{x}}_{A}\right)$ is its associated correlator. Note that *c*(*A*) is known as a spin-correlator in many-body physics and is, up to a factor of $1/2^{|A|}$, equivalent to the Fourier coefficient associated with the parity function χ(A), as defined in Fourier analysis of Boolean functions (*25*).

The cumulants κ(S) required for the sampling procedure are computed from the correlators *c*(*S*) for all subsets *S* of size 1 to *K* of [*M*] using the relation

$$\kappa \left(S\right)=\sum_{\pi \in P\left(S\right)}{\left(-1\right)}^{|\pi |-1}\left(|\pi |-1\right)!\prod_{B\in \pi }c\left(B\right)$$ (2)

with P(S) being the set of all partitions of *S*. The computation of the correlators is the only aspect of our algorithm that depends on the peculiarities of GBS. In section S2, we show how these correlators of order *d* can be reexpressed analytically as a large sum of Gaussian integrals, leading to the expression

$$c\left(S\right)={\left(-1\right)}^{|S|}\sum_{R\subseteq S}\frac{{\left(-2\right)}^{|R|}}{\sqrt{\text{det}\left[\frac{1}{\hbar }\left(\sigma_{R}+\frac{\hbar }{2}I\right)\right]}}$$ (3)

where $\sigma_{R}$ is the 2R×2R covariance matrix of the subsystem *R*. It is easy to see that the computation time for each correlator of order *d* scales as $O\left(d^{3}2^{d}\right)$ as it requires the computation of 2*^d^* determinants of size at most *d*. In addition, the computation time for each cumulant of order *d* scales as $O\left({\mathrm{dB}}_{d}\right)$, where $B_{d}$ is the *d*th Bell number equaling the number of partitions of a set of size *d*, which compounds the complexity to $O\left(d{\left(d/\left(e\text{ln}d\right)\right)}^{d}\right)$. As we restrict to a small truncation order *K*, each term is extremely fast to compute, and the dominant factor in the scaling comes from the total number of correlators $\left(\begin{array}{c} M\\ K \end{array}\right)\approx O\left(M^{K}\right)$.

#### Sampling algorithm

We are interested in sampling from a GBS distribution of *M* binary outcomes. The classical algorithm we construct is based on the chain rule of conditional probabilities $p\left(x_{n}|x_{n-1},\ldots ,x_{1}\right)$. Central to our algorithm is an expression for the bias $\Delta_{x_{n}}=p\left(0,x_{n-1},\ldots ,x_{0}\right)-p\left(1,x_{n-1},\ldots ,x_{0}\right)$ between the outputs 0 and 1 of the *n*th bit. The expression, presented in more detail in section S2, is a collection of terms containing cumulants of increasing order

$$\Delta_{x_{n}}=\sum_{R\subseteq \left[n-1\right]}\frac{1}{2^{|R|}}\chi \left(R\right)\kappa \left(R\cup \left\{n\right\}\right)\;p\left(\bar{R}\right)$$ (4)

where κ(R∪{n}) is the cumulant of order ∣R∣+1 associated with the union of the singleton {n} containing the index of the bit to be sampled and a subset *R* of indices of bits sampled in the past, χ(R∪{n}) is its associated parity function, and $p\left(\bar{R}\right)$ is the marginal probability over the complement of *R* ($\bar{R}=\left[n-1\right]\backslash R$). Note that the term R=∅ ∅ in the sum of Eq. 4 corresponds to the *n*th output being completely independent of all previous bits and fully determined by κ({n})=c({n}), while the subsequent terms in the sum add correlation with previous bits $x_{1}$ to $x_{n-1}$. From the value of $\Delta_{x_{n}}$ and the previously computed probability $p\left(x_{n-1},\ldots ,x_{1}\right)$, one can sample the *n*th bit and compute $p\left(x_{n},\ldots ,x_{1}\right)$.

Taking the sum over all partitions of [*n*] into consideration would introduce a costly $O\left(2^{n}\right)$ scaling. It is often the case that local noise in large quantum circuits has the effect of strongly suppressing high-order correlations and cumulants. For example, an inspection of previous GBS experimental data shows that the value of cumulants beyond fifth order is statistically insignificant (*3*–*6*), and the cumulants estimated from the samples of the high-power Jiuzhang 2.0 and 3.0 experiments were shown to have negligible correlation to their corresponding ground truth cumulants at sixth order in (*18*). This justifies truncating the sum in Eq. 4 to a constant order K (∣R∣+1≤K). The truncated version of $\Delta_{x_{n}}$ requires computing $\sum_{j=1}^{K}\left(\begin{array}{c} n\\ j \end{array}\right)\approx O\left(n^{K}\right)$ terms, each consisting of a cumulant, a parity function, and a marginal $p\left(\bar{R}\right)$. With the dominant contribution coming from the last sampled bit, we have $O\left(M^{K}\right)$ terms in the truncated version of Eq. 4.

The $O\left(M^{K}\right)$ required cumulants have been computed in the preprocessing stage using the definition presented in the “Precomputing the cumulants” section. The parity functions are simple to compute with simple bitwise multiplication and additions. The only nontrivial objects to compute are the marginals $p\left(\bar{R}\right)$. Despite $\bar{R}$ matching previously appearing variables in the chain rule, $p\left(\bar{R}\right)$ cannot be directly computed from previous data, as we would need to sum over variables *R* for values that never occurred. In addition, the positions of these *R* elided variables change with each term of the sum. This makes the exact calculation of $p\left(\bar{R}\right)$ prohibitively expensive. The key aspect of constructing our algorithm is to design good approximations to the marginals $p\left(\bar{R}\right)$. We present a pedagogical, simplified example in Materials and Methods and provide full detail in section S3. In short, our algorithm can be understood as a dynamical program where we keep track of a series of tables, one for each family of approximated marginals and one for the sampling probabilities $p\left(x_{n},\ldots ,x_{1}\right)$. At each step, the tables are updated from a nontrivial combination of previously computed values and precomputed cumulants following specific rules inspired by an adaptation of Eq. 4 to marginal distributions.

### Comparison to experiments

In this section, we will compare the samples generated by our sampler together with the samples generated by different 144-mode Jiuzhang experiments, focusing on cases that were also analyzed in (*18*). In addition, in section S5, we show that our sampler performs essentially equivalently to ref. (*18*). Throughout, we will use the convention established in (*18*) for naming the different Jiuzhang configurations. All emulator samples were generated using the “double-elision” algorithm at order *K* = 5, described in section S3. In each case, we generated 10 million samples in 115 min using eight GPUs on a cluster node.

Before presenting the experimental results, we note that the gold standard for comparing probability distributions—the total variation distance between an experiment and a sampler or ground truth—is infeasible in regimes where a quantum advantage is claimed. Computing the full distribution is classically intractable, and estimating it experimentally is equally impractical. Given these limitations, we follow standard practice and use the metrics described below. Last, note that we do not benchmark against Jiuzhang 1.0 given that most of the evidence for quantum advantage in this experiment has been shown to be reproducible using unentangled Gaussian states (so-called squashed states) (*26*).

#### Validation on low brightness

Figure 2 shows our performance results against a low-brightness configuration of the Jiuzhang 2.0 experiment, the configuration J2-P65-2 with an output photon-number density of ν=0.093. Because of the large number of modes in the system, verifying the proximity between the sampler’s distribution and the ground truth of the experiment using the total variation distance, a standard statistical metric, cannot be done efficiently. Nevertheless, the low brightness of this configuration allows us to use XEB as a sample-efficient measure of similarity between the sampler’s distribution and the ground truth as well as the calculation of click cumulants up to fifth order (see sections S6.1 and S6.3 for the description of the XEB score and click cumulants), which have been widely used verification techniques for recent implementations of GBS and the recent classical emulation in (*18*).

**Fig. 2.**
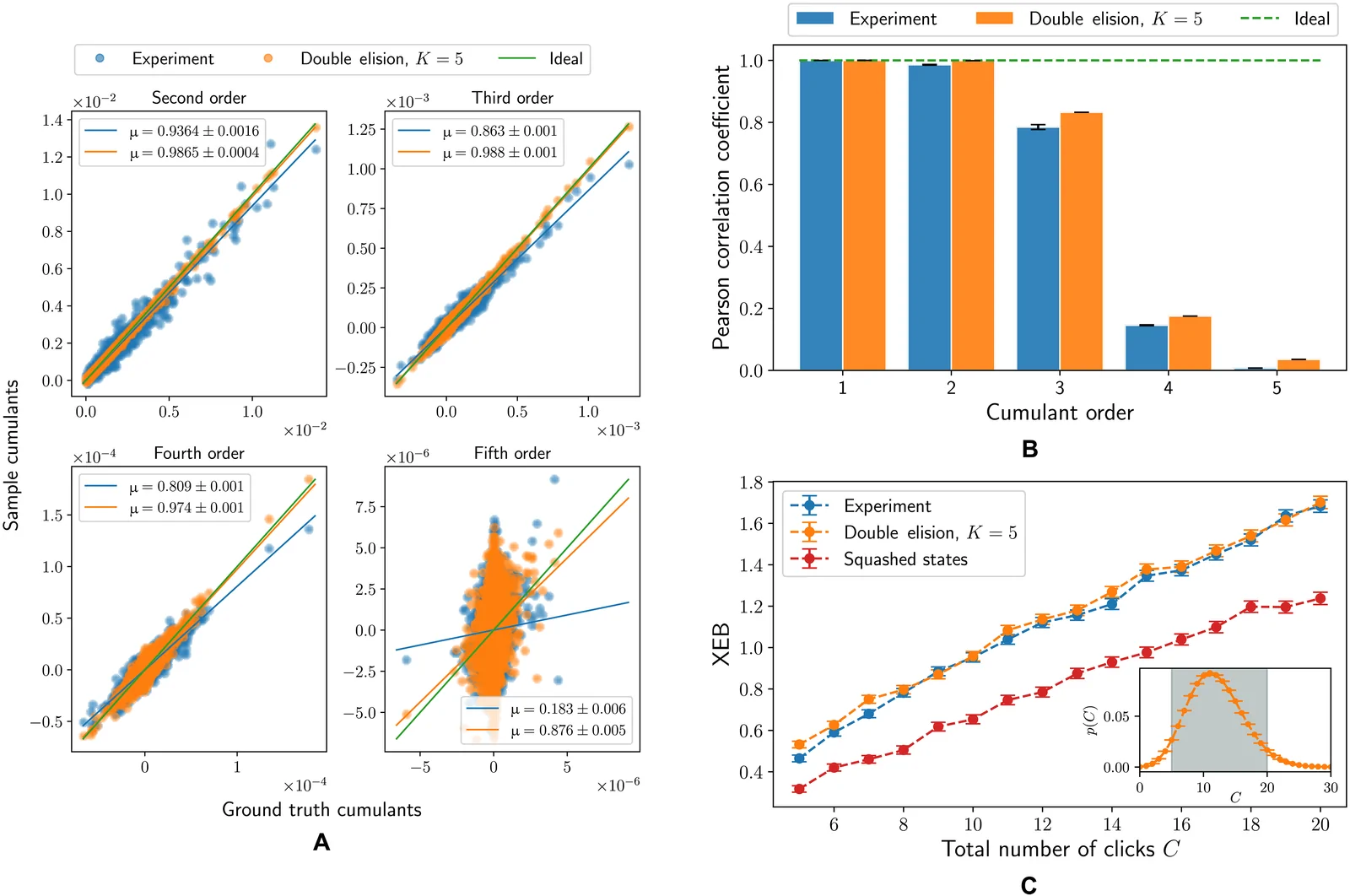
Validation tests for the J2-P65-2 experiment. (**A**) Comparison between the estimated click cumulants (orders 2 to 5), from experimental (blue circles) and double-elision samples with *K* = 5 (orange circles), and those predicted by the ground truth of the experiment (green line). The vertical axis corresponds to cumulants obtained from samples, either experimental or double elision, while the horizontal axis corresponds to the ground truth (i.e., theoretical) cumulants. The blue and orange lines represent the linear fit between sample cumulants and those from the ground truth. The slope of each linear fit is shown in the legends. All cumulants were estimated using 10^7^ samples. (**B**) Pearson correlation coefficients between sample and ground truth cumulants. The green dashed line indicates the ideal value of these coefficients, which is equal to 1. Error bars were obtained using 100 bootstrapping resamples. (**C**) XEB as a function of the total number of clicks *C* for both experimental (blue dashed line with circles) and double-elision (orange dashed line with circles) samples. For reference, we also include the XEB scores of the squashed states model (red dashed line with circles) (*26*). Error bars were obtained through error propagation. The estimation of the XEB scores used 4000 samples per number of clicks. The inset figure shows the total click distribution, *p*(*C*), of the sampler. The shaded region indicates the range of total number of clicks, *C*, for which the XEB scores were computed.

The scatterplots in Fig. 2A show the comparison between the estimated cumulants (orders 2 to 5) from both experimental and double-elision samples and those predicted by the ground truth of the experiment. We estimated the cumulants using 10^7^ experimental and double-elision samples. The linear fits between sample and theoretical cumulants show that the emulator cumulants are better correlated with the theoretical values than those estimated from the experimental samples. The slopes μ of the linear fits between double-elision and ground truth cumulants are closer to the ideal value of μ = 1 than those of the experimental cumulants. This is further confirmed by the Pearson correlation coefficients *r* depicted in Fig. 2B: Our sampler’s correlation coefficients are higher and closer to the ideal value of *r* = 1.

In Fig. 2C, we show the XEB score as a function of the total number of clicks, *C*, for both experimental and double-elision samples. For reference, we also show the XEB scores for a squashed states model, which has previously been considered as a possible explanation for the Jiuzhang 2.0 experiment (*26*). Each value of the XEB score was estimated using 4000 samples. We computed the XEB scores in the range 5≤C≤20, which, according to the inset of Fig. 2C, represents the most important part of the total click distribution of J2-P65-2. As can be seen, the double-elision algorithm closely reproduces the XEB scores and, for some values of *C*, outperforms the experimental samples’ scores.

#### High-brightness results for Jiuzhang 2.0

We now move on to the validation of our sampler for a Jiuzhang 2.0 configuration with high brightness, namely the J2-P65-5 configuration with ν=0.975. For this setup, the most relevant part of the total click distribution lies at C>40, making the XEB test too costly to carry out on the statistically relevant click counts. Similarly, as in (*18*), we will rely solely on the comparison of click cumulants and the comparison of the total click distribution to investigate the performance of our sampler.

In Fig. 3A, we show the comparison between sample and ground truth cumulants (orders 2 to 5), as well as the corresponding linear fits. We computed the cumulants using 10^7^ experimental and double-elision samples. Again, we can readily see that, to all orders, our emulator’s cumulants are better correlated with the ground truth than those estimated from the experimental samples. This is also confirmed by the Pearson correlation coefficients shown in Fig. 3B. Last, Fig. 3C shows a comparison of the total click distribution as a function of *C*. We can see that the distribution of the double-elision algorithm neatly overlaps with the ground truth distribution, while that of the experimental outcomes is slightly shifted.

**Fig. 3.**
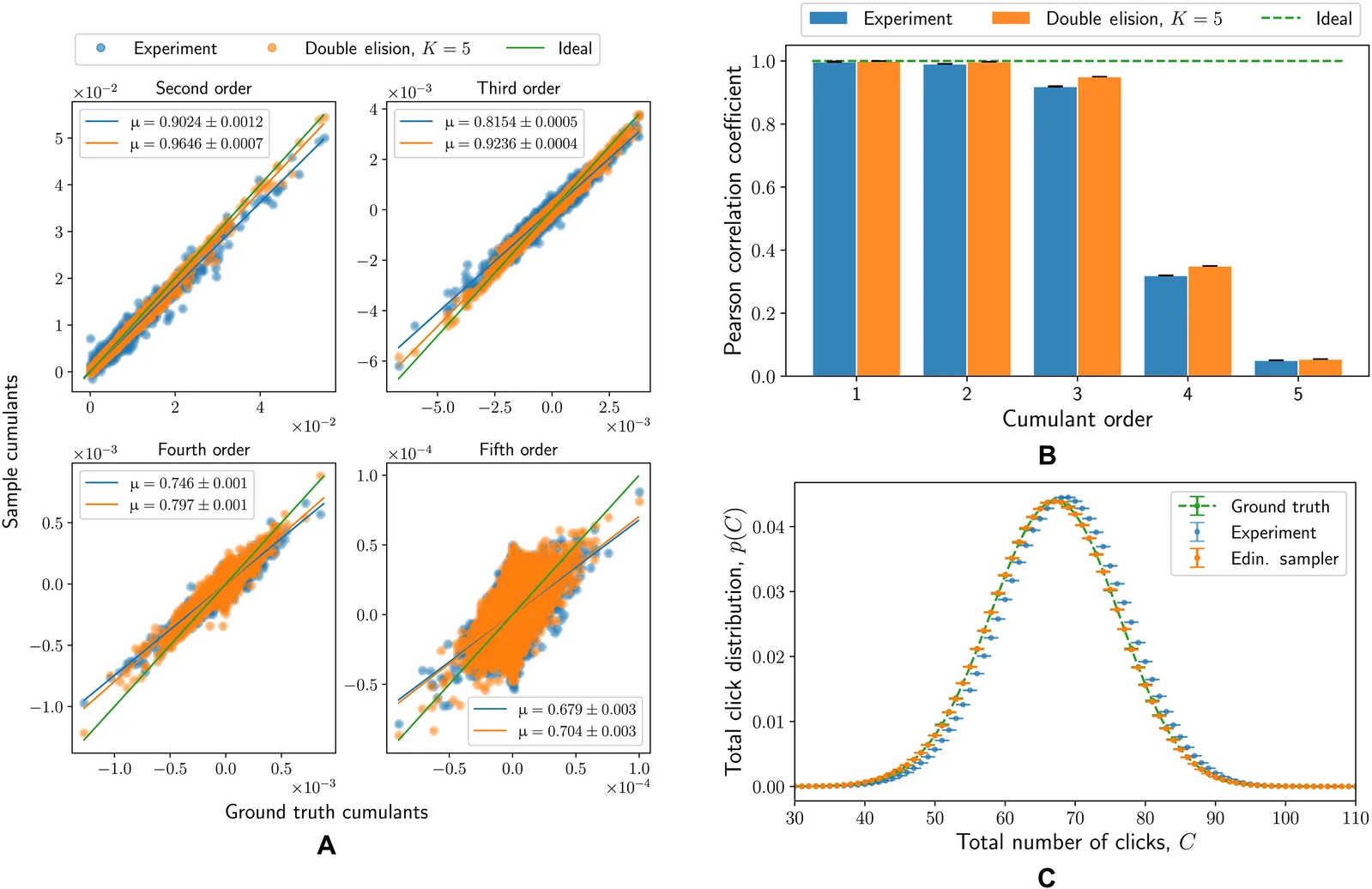
Validation tests for the J2-P65-5 experiment. (**A**) Comparison between the estimated click cumulants (orders 2 to 5), from experimental (blue circles) and double-elision samples with *K* = 5 (orange circles), and those predicted by the ground truth of the experiment (green line). The vertical axis corresponds to cumulants obtained from samples, either experimental or double-elision, while the horizontal axis corresponds to the ground truth (i.e., theoretical) cumulants. The blue and orange lines represent the linear fit between sample cumulants and those from the ground truth. The slope of each linear fit is shown in the legends. All cumulants were estimated using 10^7^ samples. (**B**) Pearson correlation coefficients between sample and ground truth cumulants. The green dashed line indicates the ideal value of these coefficients, which is equal to 1. Error bars were obtained using 100 bootstrapping resamples. (**C**) Total click distribution as a function of the total number of clicks *C* for both experimental (blue circles) and double-elision (orange circles) samples. The ground truth distribution corresponds to the green dashed line with circles, which was obtained using phase space techniques (*33*). Error bars were obtained through bootstrapping.

#### High-brightness results for Jiuzhang 3.0

We also validate the performance of our sampler against the “high-power” configuration of the Jiuzhang 3.0 experiment, i.e., J3-high, with a photon-number density of ν = 0.81. In this experiment, the input light propagates through a lossy interferometer with 144 spatial modes and is then time-domain multiplexed into eight bins for readout using threshold detectors to implement a pseudo–photon number resolution. This implies that the actual Gaussian state before measurement has *M* = 1152 modes rather than *M* = 144. Nevertheless, following a similar strategy as the one proposed in (*18*), we will verify our sampler against a “coarse-grained” configuration with *M* = 144. As in the case of J2-P65-5, the computation of the XEB scores becomes too costly in the relevant range of the total number of clicks, C>40. Thus, we will focus on the comparison of click cumulants and distribution of the total number of clicks. Figure 4 shows that the performance of our sampler for these coarse-grained tests is as good as or better than the experiment.

**Fig. 4.**
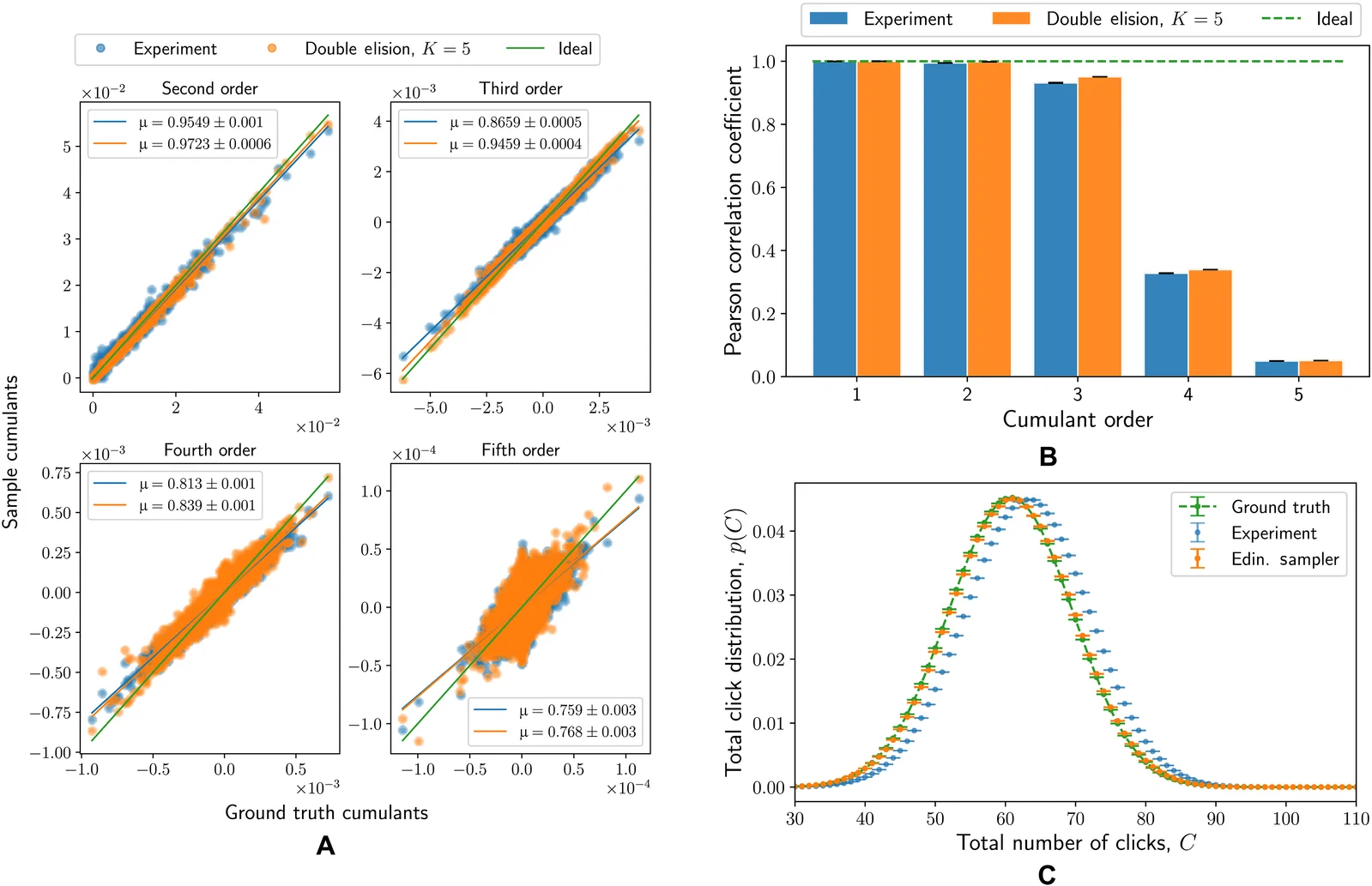
Validation tests for the J3-high experiment. (**A**) Comparison between the estimated click cumulants (orders 2 to 5), from experimental (blue circles) and double-elision samples with *K* = 5 (orange circles), and those predicted by the ground truth of the experiment (green line). The vertical axis corresponds to cumulants obtained from samples, either experimental or double-elision, while the horizontal axis corresponds to the ground truth (i.e., theoretical) cumulants. The blue and orange lines represent the linear fit between sample cumulants and those from the ground truth. The slope of each linear fit is shown in the legends. All cumulants were estimated using 10^7^ samples. (**B**) Pearson correlation coefficients between sample and ground truth cumulants. The green dashed line indicates the ideal value of these coefficients, which is equal to 1. Error bars were obtained using 100 bootstrapping resamples. (**C**) Total click distribution as a function of the total number of clicks *C* for both experimental (blue circles) and double-elision (orange circles) samples. The ground truth distribution corresponds to the green dashed line with circles and was obtained using phase space techniques (*33*). Error bars were obtained through bootstrapping. The estimation of the experimental and double-elision total click distributions was obtained using 10^7^ samples.

### Cost estimation and scaling

In what follows, we present results that demonstrate the scaling of the different computational tasks of our procedure and its capability for parallelization. We used a CPU for this analysis, as we could explicitly run the tools with various numbers of cores. For a general use, a GPU implementation, as used in the “Comparison to experiments” section, is recommended for a greater sampling speed.

As discussed in the “Emulator description” section, our sampling algorithm should scale as $O\left(M^{K}\right)$ in time and space, where *M* is the number of modes in the Gaussian state, and *K* is the fixed truncation order. Figure 5A, using a log-log scale, shows the sampling time of the algorithm as a function of the number of modes for truncation orders *K* = 4 and *K* = 5, from which we infer good agreement with polynomial scaling $O\left(M^{K}\right)$. The power is slightly higher for *K* = 4 and lower for *K* = 5, which is potentially an implementation artifact. Figure 5B shows the sample generation rate as a function of the number of cores used. The clear linear relation for both *K* = 4 and *K* = 5, albeit with different slopes, demonstrates the embarrassingly parallelizable nature of the algorithm.

**Fig. 5.**
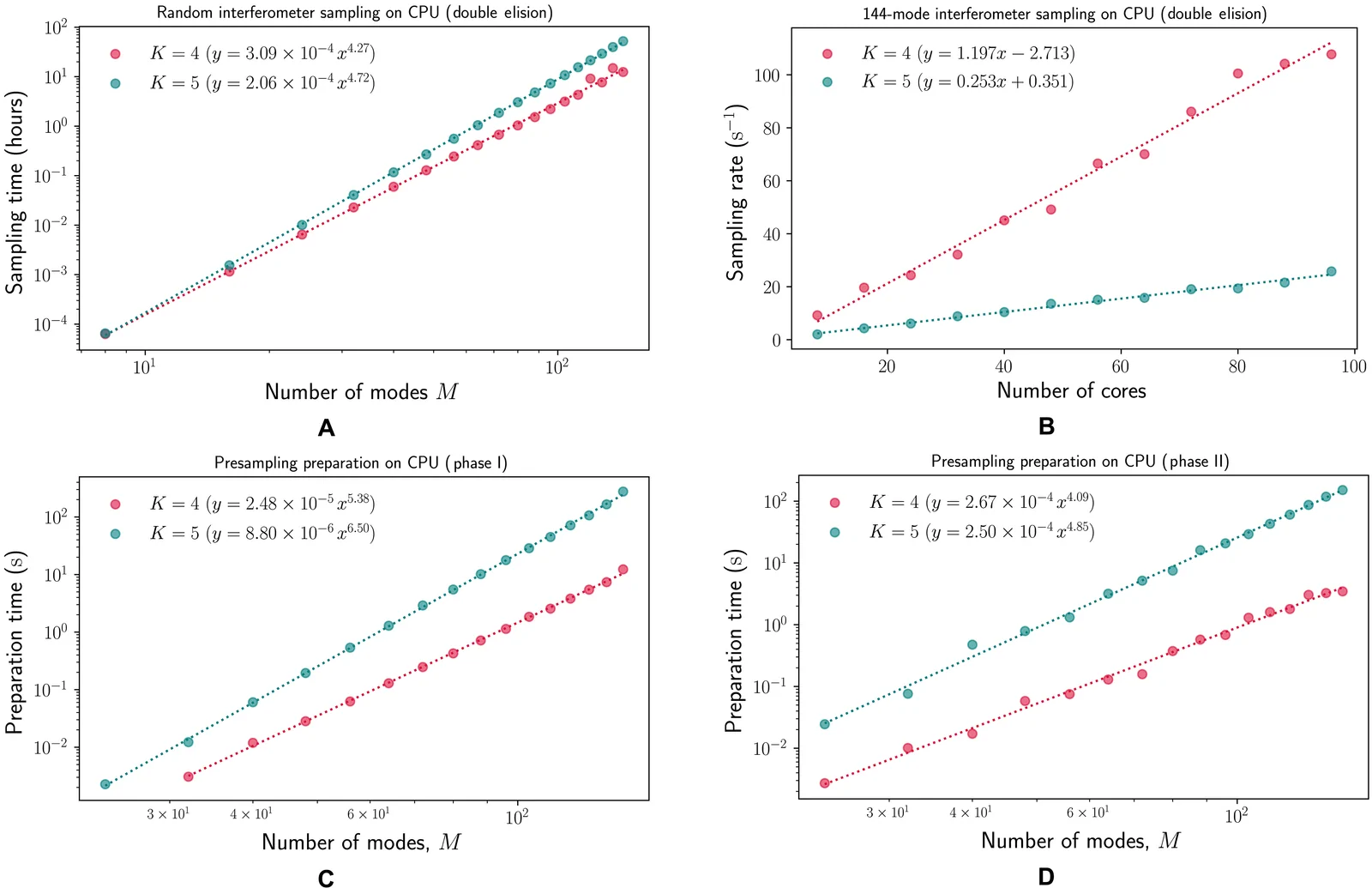
Double-elision algorithm cost estimation. (**A**) Sampling time (in hours) as a function of the number of modes, *M*, for *K* = 4 (red circles) and *K* = 5 (teal circles). Each point indicates the time it takes to generate 10^7^ samples. (**B**) Sampling rate (in s^−1^) as a function of the number of cores for *K* = 4 (red circles) and *K* = 5 (teal circles). (**C**) Time of phase I (in s), i.e., data structure preprocessing, as a function of the number of modes, *M*, for *K* = 4 (red circles) and *K* = 5 (teal circles). (**D**) Time of the phase II presampling preparation (in s) as a function of the number of modes, *M*, for *K* = 4 (red circles) and *K* = 5 (teal circles). In (A) to (D), the dotted lines correspond to the linear or power law fits shown in the legends. In (A), (C), and (D), both the horizontal and vertical axes are in logarithmic scale. All data were obtained using randomly generated interferometers, except for (B) where we used the 144-mode interferometer of the J3-high configuration.

The preprocessing stage discussed in the “Precomputing the cumulants” section precomputes the correlators and cumulants of a given ground truth, which are later used during the generation of samples. This procedure consists of two phases. The first phase is a data structure preprocessing task that only needs to be run once for each choice of *M* and *K*, i.e., it does not change as we change the GBS circuit. It relates to Eq. 2 and consists of obtaining all the sets of indices of the form $S=\left\{i_{1},\ldots ,i_{d}\right\}\subseteq \left[M\right]$, with *d* up to *K*, as well as on the computation of all necessary “partition patterns,” which are used to identify any partition of a given *S* in Eq. 2. Each pattern corresponds to a partition π of the set {1,…,d} and a constant $w\left(\pi \right)={\left(-1\right)}^{|\pi |-1}\left(|\pi |-1\right)!$ that determines the contribution of that partition to the estimation of the cumulants in the next phase of preprocessing. We remark that in the case of the statistical estimation of cumulants from sampled data used in our click cumulant analysis, the weights w(π) have an extra correction factor depending on the number of samples [see section S6.1 and (*27*)].

The second preprocessing stage consists of computing all the cumulants of a given GBS up to order *K* using Eq. 3. Each block b∈π is mapped to a correlator $c\left(\{i_{k}\;\text{with}\;k\in b\}\right)$. Then, these correlators are multiplied together according to π, weighted, and summed to obtain the necessary cumulants following Eq. 2. Figure 5 (C and D) shows the time complexity of phases I and II, respectively. Although phase II approximately follows the expected time complexity of $O\left(M^{K}\right)$, phase I scales more like $O\left(M^{K+1}\right)$ or higher, which potentially hints at the need for an improvement in its implementation.

The space complexities of the preprocessing and sampling algorithm are more straightforward to analyze. The presampling stage requires just enough memory to compute and store the cumulants subsequently used by the sampling algorithm. Although there are $\left(\begin{array}{c} M\\ K \end{array}\right)\approx O\left(M^{K}\right)$ cumulants in total, our implementation for computing the cumulants from the correlators also requires the use of an array of auxiliary indices of size $O\left({\mathrm{KM}}^{K}\right)$. For sampling, there are two dominant sources of memory cost: the precomputed array of $O\left(M^{K}\right)$ cumulants and the dynamically updated table of $O\left(M^{3}\right)$ approximated marginals during the generation of a sample. For the 144-mode algorithm at *K* = 5, the array of cumulants requires 2 gigabytes, the auxiliary indices require 2.5 gigabytes, and the table of marginals while sampling requires 2 megabytes per thread.

## DISCUSSION

We have proposed a GBS emulator based on an expansion in terms of cumulants up to a chosen truncation order. Its implementation is embarrassingly parallelizable and benefits from polynomial scaling in the system size, with an exponent depending on the truncation order. We believe that its characteristics make it a solid contender against future generations of GBS hardware. We also believe that the algorithm is easily generalizable to photocounting.

Our work shows that a strong requirement for hardness of GBS is the presence of nontrivial high-order cumulants, not just in the original noiseless distribution but more importantly in the experimental implementation affected by losses and other imperfections. Therefore, hardware design should be oriented at carefully balancing the increase in modes while preserving or increasing the high-order correlations, as low-order emulators are not difficult to implement and scale, as we have shown in this work.

Losses and imperfections are not directly exploited in our algorithm’s design beyond motivating the truncation of the cumulant expansion. Therefore, our algorithm would be able to simulate noiseless quantum circuits that have negligible cumulants beyond a constant order *K*, something that would not be possible with other emulators of GBS.

High-brightness sources impose surmountable challenges to our emulator, as they potentially increase the cumulant expansion truncation order and also require better marginal approximations resulting from the larger cumulant values. One should not immediately infer from this that increasing the brightness is a good strategy to preserve the hardness of simulation. We believe that adapting our algorithms to decomposition of highly noisy states in terms of displaced pure states of low brightness, as done in (*18*), will support our intuition.

To scale the number of modes into the thousands to simulate the very recent Jiuzhang 4.0 experiment (*7*), we may need a new implementation that not only makes use of multiple nodes of a high-performance computing cluster but also accurately handles large sums of extremely small probabilities. At the same time, future algorithmic improvements, such as the decomposition of a highly noisy state in terms of displaced pure states or exploiting the structure of the loop-architecture circuits, could further downscale the costs. One may also expect a slightly more efficient computation of the Fourier coefficients, as was already achieved for Torontonians (*28*, *29*), or a direct computation of the cumulants, avoiding the intermediate calculation of joint moments, as is known for photon-number cumulants (*30*).

We leave obtaining theoretical guarantees of how close our sampler is to the actual ground truth distribution for future work. Ideally, one would like to have a clear relation between the required levels of truncation *K*, as a function of the characteristic of the GBS sampler, such as losses, squeezing, and number of modes. A necessary step in this direction is to get theoretical bounds or asymptotics on the decay of cumulants as a function of *K*, losses, and number of modes, usually averaged over random interferometers; numerical evidence for this decay has been presented in (*30*), and analytical results in the same direction will be presented elsewhere using Weingarten calculus (*31*). Unfortunately, an analogous calculation for on-off detectors is significantly harder as the associated cumulants are not polynomials in the entries of the unitary matrix representing the interferometer (*28*, *32*).

Our algorithmic framework based on a cumulant expansion and approximated marginals is valid for any binary output probability distribution, rendering it applicable beyond the emulation of GBS. For example, it could be adapted to emulate qubit-based quantum circuits, such as IQP and QAOA. To be applicable, the noiseless or noisy circuit needs to satisfy the following: (i) Its cumulants of order higher than a constant *K* are negligible, and (ii) its cumulants can be efficiently computed up to order *K*. For example, one could envision using our tools to dequantize “train classically deploy quantum” proposals. In cases where assumption (i) above holds but not (ii), one could still design classical surrogate models of quantum circuits. These are families of quantum-enhanced classical algorithms where samples from a quantum device are used to train a classical model (estimate the cumulants) that learns to replicate the behavior of the quantum device using purely classical resources (our sampling algorithm). Last, our work highlights the need to provide proper complexity-theoretic benchmarks for bosonic quantum advantage. Partial progress has been reported in (*23*) for diluted and undiluted (*24*) photon-number density but is so far limited to photon-number distributions, with Haar random interferometers.

## MATERIALS AND METHODS

### Marginal approximations

As a pedagogical example, we present a simplified “single-elision” variant of the sampling algorithm at *K* = 3 that has a reduced time complexity and space complexity of $O\left(M^{3}\right)$. The double-elision algorithm used to obtain the results in the “Validation on low brightness” section is presented in full detail in section S3.

The algorithm can be understood as a dynamical program, where we keep track of three tables. The first table stores the auxiliary partial marginals $p^{\left(+\right)}\left(x_{a},\ldots ,x_{b}\right)$, containing all variables between $x_{a}$ and $x_{b}$, where a>b and a,b∈[n−1]. The second table stores the auxiliary “elided” marginals $p^{\left(1\right)}\left(x_{a},\ldots ,{\tilde{x}}_{e},\ldots ,x_{1}\right)$, containing all variables between $x_{1}$ and $x_{a}$, except for the variable $x_{e}$ (we use ${\tilde{x}}_{e}$ to denote that the variable is missing in the marginal), where, similarly, a∈[n−1]. Last, the third table holds all of the sampling probabilities $p\left(x_{a},\ldots ,x_{1}\right)$, where, again, a∈[n−1]. With each generated bit, these tables are updated using a nontrivial combination of previously filled values and the precomputed cumulants.

Following Eq. 4, the joint probability $p\left(x_{n},\ldots ,x_{1}\right)$ of the first *n* bits can be expanded up to order *K* = 3

$$\begin{array}{c} p\left(x_{n},\ldots ,x_{1}\right)\\ =\frac{1}{2}\left(1+\gamma_{\left\{n\right\}}\right)\;p\left(x_{n-1},\ldots ,x_{1}\right)\\ +\frac{1}{4}\sum_{i=1}^{n-1}\gamma_{\left\{i,n\right\}}p^{\left(1\right)}\left(x_{n-1},\ldots ,{\tilde{x}}_{i},\ldots ,x_{1}\right)\\ +\frac{1}{8}\sum_{\begin{array}{c} i,j=1\\ i>j \end{array}}^{n-1}\gamma_{\left\{i,j,n\right\}}p^{\left(+\right)}\left(x_{n-1},..,x_{i+1}\right)\;p^{\left(1\right)}\left(x_{i-1},..,{\tilde{x}}_{j},..,x_{1}\right) \end{array}$$ (5)

where each $\gamma_{S}$ is the product of the order-|*S*| cumulant κ(S) and the associated parity function $\chi_{S}\left(\bar{x}\right)$, and we only include the terms in the second sum for which i>j.

The second-order term in Eq. 5 has marginals with a single missing variable, which we approximate via the auxiliary marginal distribution

$$\begin{array}{c} p^{\left(1\right)}\left(x_{n},\ldots ,{\tilde{x}}_{e},\ldots ,x_{1}\right)\\ =\frac{1}{2}\left(1+\gamma_{\left\{n\right\}}\right)\;p^{\left(1\right)}\left(x_{n-1},\ldots ,{\tilde{x}}_{e},\ldots ,x_{1}\right)\\ +\frac{1}{4}\sum_{\begin{array}{c} i=1\\ i\neq e \end{array}}^{n-1}\gamma_{\left\{i,n\right\}}p^{\left(+\right)}\left(x_{n-1},..,x_{b+1}\right)\;p^{\left(1\right)}\left(x_{b-1},..,{\tilde{x}}_{a},..,x_{1}\right) \end{array}$$ (6)

where a=min(i,e), and b=max(i,e); we exclude the term in the sum for which i=e and the term *i* = 1 has $p^{\left(1\right)}$ replaced by a second $p^{\left(+\right)}$, as we elide the first variable. The third-order term in Eq. 5 does not contain a double-elided auxiliary marginal $p^{\left(2\right)}\left(x_{n-1},\ldots ,{\tilde{x}}_{b},\ldots ,{\tilde{x}}_{a},\ldots ,x_{1}\right)$, as computing these would lead to scaling in the time complexity beyond $O\left(M^{3}\right)$. A similar case can be made for the second-order term in Eq. 6. We therefore use a cheaper approximation of those marginal terms via a product of two independent terms, one of which is the auxiliary partial marginal

$$\begin{array}{c} p^{\left(+\right)}\left(x_{n},\ldots ,x_{\ell }\right)\\ =\frac{1}{2}\left(1+\gamma_{\left\{n\right\}}\right)\;p^{\left(+\right)}\left(x_{n-1},\ldots ,x_{\ell }\right)\\ +\frac{1}{4}\sum_{i=\ell }^{n-1}\gamma_{\left\{i,n\right\}}p^{\left(+\right)}\left(x_{n-1},\ldots ,x_{i+1}\right)\;p^{\left(+\right)}\left(x_{i-1},\ldots ,x_{\ell }\right) \end{array}$$ (7)

where the term i=ℓ has only the left $p^{\left(+\right)}$ contribution. The *n*th step of computing $p\left(x_{n},\ldots ,x_{1}\right)$ from previously estimated values involves a sum of $O\left(n^{2}\right)$ terms. For a bitstring of size *M*, the time complexity is $O\left(M^{3}\right)$. A similar argument shows that computing all $p^{\left(+\right)}\left(x_{n},\ldots ,x_{\ell }\right)$ and $p^{\left(1\right)}\left(x_{n},\ldots ,{\tilde{x}}_{e},\ldots ,x_{1}\right)$ values has the same total time complexity of $O\left(M^{3}\right)$. These auxiliary marginals add a limited cost of storing $O\left(M^{2}\right)$ additional values related to the auxiliary marginal tables for each concurrent thread alongside the global $O\left(M^{3}\right)$ requirement of storing the cumulants up to order *K* = 3.

Because of the exclusion of higher-order cumulants and approximations of nested marginal expressions, the computed probabilities can occasionally be smaller than zero or more than one, in which cases their values are clamped to be equal to zero or one, respectively. The rates at which this occurred for *p*, $p^{\left(+\right)}$, $p^{\left(1\right)}$, and $p^{\left(2\right)}$ while generating J3-high samples were 0.04, 0.2, 0.7, and 0.9%, respectively.

### Implementation

For the statistical comparison to each experiment, we generated 10 million samples in 115 min using eight Nvidia H100 SXM 80-gigabyte GPUs on one cluster node. Each GPU used 2 gigabytes of memory to store the list of cumulants up to fifth order, and the rest of the available memory was split into units of 2 megabytes to hold each launched thread’s tables of marginals (each thread was responsible for generating a single sample). Each kernel run generated a batch of samples with a size determined by the available memory, which, in this case, was ∼38,000. To generate the complete list of samples, the kernel was launched multiple times across each GPU.

The benchmarks were obtained using a single AMD EPYC 9654 CPU of Calcul Québec’s Rorqual compute cluster. For the cost estimation and scaling tests, we used a CPU implementation multithreaded with OpenMP. The threads read from a single shared list of cumulants, again requiring 2 gigabytes of memory, and each used a private 2-megabyte cache to store the tables of marginals required for the current sample. Unlike the GPU implementation, each thread is responsible for iteratively generating multiple samples rather than just one, and the memory storing the tables of marginals can easily be continually reused.

## Supplementary Materials

### This PDF file includes:

Supplementary Text Table S1 Figs. S1 and S2 References
