# Supplementary material for: A fast and frugal Gaussian boson sampling emulator
Source: Sci Adv. 2026 Oct 14;12(42):eaef8431. doi: 10.1126/sciadv.aef8431 (PMC13665930; doi:10.1126/sciadv.aef8431)
Supplement: Supplementary file 1 [file sciadv.aef8431_sm.pdf]

Science Advances
AAAS

Supplementary Materials for

A fast and frugal Gaussian boson sampling emulator

Tom Dodd *et al.*

Corresponding author: Raúl García-Patrón, rgarcia3@ed.ac.uk

Sci. Adv. **12**, eaef8431 (2026)
DOI: 10.1126/sciadv.aef8431

This PDF file includes:

Supplementary Text
Table S1
Figs. S1 and S2
References

1 Probabilities, Fourier coefficients and cumulants

A probability distribution $p(\bar{x})$ over the sample space of n binary variables, $\bar{x} = (x_1, x_2, \ldots, x_n)$, is a non-negative Boolean function, $p(\bar{x}) : \{0,1\}^n \to \mathbb{R}$ with an input *bitstring* of length n and a real-valued output encoding the corresponding occurrence probability. Analysis of Boolean Functions (*25*) shows that $p(\bar{x})$ belongs to a Hilbert space of dimension 2^n with inner product given by

$$\langle p(\bar{x}), q(\bar{x}) \rangle = \frac{1}{2^n} \sum_{\bar{x} \in \{0,1\}^n} p(\bar{x}) q(\bar{x}). \tag{S1}$$

The parity functions

$$\chi_{\bar{s}}(\bar{x}) = (-1)^{\bar{s} \cdot \bar{x}}, \tag{S2}$$

indexed by the bitstring $\bar{s} \in \{0,1\}^n$, where $\bar{s} \cdot \bar{x} = \sum_{k=1}^{n} x_k s_k$, form a basis of the Hilbert space and satisfy the orthogonality condition

$$\sum_{\bar{x} \in \{0,1\}^n} \chi_{\bar{s}}(\bar{x}) \chi_{\bar{s}'}(\bar{x}) = 2^n \delta_{\bar{s},\bar{s}'}. \tag{S3}$$

Using this basis, we can expand the probability distribution as

$$p(\bar{x}) = \sum_{\bar{s} \in \{0,1\}^n} \hat{p}(\bar{s}) \chi_{\bar{s}}(\bar{x}), \tag{S4}$$

where the $\hat{p}(\bar{s}) = \langle p(\bar{x}), \chi_{\bar{s}}(\bar{x}) \rangle$ are known as *Fourier coefficients*. The following inverse relation also holds

$$\hat{p}(\bar{s}) = \frac{1}{2^n} \sum_{\bar{x} \in \{0,1\}^n} p(\bar{x}) \chi_{\bar{s}}(\bar{x}) \tag{S5}$$

The quantity $|\bar{s}| = \sum_{i=1}^{n} s_i$ represents the *Hamming weight* of the Fourier coefficient.

Note that every $\bar{s}$ is uniquely determined by the number of 1's it has (i.e. its Hamming weight) and by their positions within the bitstring. These positions can be parametrized by a subset $S \subseteq [n] = \{1, \ldots, n\}$, while the Hamming weight is simply given by the cardinality of the subset, noted $|S|$. This allows us to associate every $\bar{s} \in \{0,1\}^n$ to a unique element of $\Omega([n])$, the power set of $[n]$. On this account, we can recast Eqs. (S4) and (S5) as

$$p(\bar{x}) = \sum_{S \subseteq [n]} \hat{p}(S) \chi_S(\bar{x}) \ , \qquad \hat{p}(S) = \frac{1}{2^n} \sum_{\bar{x} \in \{0,1\}^n} p(\bar{x}) \chi_S(\bar{x}), \tag{S6}$$

where we redefine the parity functions as

$$\chi_S(\bar{x}) = (-1)^{\sum_{k \in S} x_k} = \prod_{k \in S} (-1)^{x_k}. \tag{S7}$$

1.1 Connection to spin systems

Inspecting the definition of the Fourier coefficients above, as well as that of the parity functions, we can recognize that the $\hat{p}(S)$ are, up to a 2^{-n} constant, equivalent to the definition of the *spin correlators* of a system of n spins defined by the functions $\{(-1)^{x_k}\}_{k\in S}$. The precise definition of these correlators reads

$$c(S) = \mathbb{E}\left[\prod_{k\in S}(-1)^{x_k}\right] = \sum_{\bar{x}\in\{0,1\}^n} p(\bar{x}) \prod_{k\in S}(-1)^{x_k} = 2^n \hat{p}(S), \tag{S8}$$

where $\mathbb{E}[\cdot]$ stands for the expectation value with respect to the probability distribution $p(\bar{x})$.

1.2 Joint moments, cumulants and correlators

Consider the random variables $\{x_k\}_{k\in S}$ with $S \subseteq [n]$. The joint moment of the $\{x_k\}_{k\in S}$ with respect to the distribution $p(\bar{x}) \equiv p(x_1, \ldots, x_n)$ is defined as

$$\mu(\{x_k\}_{k\in S}) = \mathbb{E}\left[\prod_{k\in S} x_k\right] = \sum_{\bar{x}\in\{0,1\}^n} p(\bar{x}) \prod_{k\in S} x_k. \tag{S9}$$

Since each of the x_k can only take values in $\{0, 1\}$, we can readily see that the joint moments can be computed as the marginal probabilities $\mu(S) = p(x_k = 1 \text{ for } k \in S)$, i.e. the probability that all bits belonging to the set S have value one.

Associated to each joint moment, we can define the cumulant (*37*) (these quantities are also known in the literature as Ursell functions (*38*), truncated correlation functions (*39*) or cluster functions (*39*)) of the $\{x_k\}_{k\in S}$ as

$$\tilde{\kappa}(\{x_k\}_{k\in S}) = \sum_{\pi\in\mathcal{P}(S)} (-1)^{|\pi|-1}(|\pi|-1)! \prod_{b\in\pi} \mu(\{x_k\}_{k\in b}). \tag{S10}$$

In this expression, π is a set partition of S, i.e. a collection of subsets of S ($\pi = \{b_1, \ldots, b_\ell\}$, $b_k \subseteq S$ for all k), usually referred to as "blocks", that are non-empty ($b_k \neq \emptyset$ for all k), mutually disjoint ($b_j \cap b_k = \emptyset$ for $j \neq k$), and whose union is equal to S ($\bigcup_{b\in\pi} b = S$). The quantity $|\pi|$ indicates the number of blocks of the partition, while $\mathcal{P}(S)$ stands for the set of all partitions of S. Relation (S10) can be inverted to obtain the joint moments as a function of the cumulants:

$$\mu(\{x_k\}_{k\in S}) = \sum_{\pi\in\mathcal{P}(S)} \prod_{b\in\pi} \tilde{\kappa}(\{x_k\}_{k\in b}). \tag{S11}$$

With these definitions in mind, we can readily see that the correlators $c(S)$ can be interpreted as the joint moments of the variables $\{(-1)^{x_k}\}_{k\in S}$, i.e. $c(S) = \mu(\{(-1)^{x_k}\}_{k\in S})$. We write the corresponding cumulants as $\kappa(S) \equiv \tilde{\kappa}(\{(-1)^{x_k}\}_{k\in S})$. Since each x_k is a binary random variable, it holds that $(-1)^{x_k} = 1 - 2x_k$, which in turn leads to the relation

$$\kappa(S) = \begin{cases} 1 - 2\mathbb{E}[x_k], & \text{for } |S| = 1, \\ (-2)^{|S|}\tilde{\kappa}(\{x_k\}_{k\in S}), & \text{for } |S| > 1, \end{cases} \tag{S12}$$

where we have used the homogeneity and translation invariance properties of the cumulants. Following this result, we can compute the correlators $c(S)$ as

$$c(S) = \sum_{\pi\in\mathcal{P}(S)} \prod_{b\in\pi} \kappa(b). \tag{S13}$$

2 Expansion of probabilities in terms of cumulants

Consider the expression for the probability distribution, $p(\bar{x}) = p(x_1, \ldots, x_n)$, in terms of the correlators $c(S)$:

$$p(x_1, \ldots, x_n) = \frac{1}{2^n} \sum_{S\subseteq[n]} \chi_S(\bar{x}) c(S). \tag{S14}$$

We want to change the sum over $S \subseteq [n]$ into a sum over $S' \subseteq [n-1]$. To do this, notice that all the subsets of $[n]$ can be divided into two groups: those that contain the element n, and those that do not. The collection of subsets that do not contain the element n corresponds precisely to the power set of $[n-1]$, while every subset of $[n]$ containing n can be written as $S' \cup \{n\}$, with $S' \subseteq [n-1]$. With these ideas in mind, we can recast Eq. (S14) as

$$p(x_1, \ldots, x_n) = \frac{1}{2^n} \sum_{S'\subseteq[n-1]} \chi_{S'}(\bar{x})\, c(S') + \frac{1}{2^n} \sum_{S'\subseteq[n-1]} \chi_{S'\cup\{n\}}(\bar{x}) c(S' \cup \{n\}). \tag{S15}$$

Note that $\chi_{S'}(\bar{x}) = \chi_{S'}(\bar{x}')$, with $\bar{x}' = (x_1, \ldots, x_{n-1})$, since it is not possible for x_n to appear in the sum $\sum_{k\in S'} x_k$ that defines $\chi_{S'}(\bar{x})$. Moreover, we have that $\chi_{S'\cup\{n\}}(\bar{x}) = (-1)^{x_n}\chi_{S'}(\bar{x}')$. Taking into account that $p(\bar{x}') = p(x_1, \ldots, x_{n-1}) = 2^{-(n-1)} \sum_{S'\subseteq[n-1]} \chi_{S'}(\bar{x}')\, c(S')$, we can write the probability distribution as

$$p(x_1, \ldots, x_n) = \frac{1}{2} p(x_1, \ldots, x_{n-1}) + \frac{(-1)^{x_n}}{2^n} \sum_{S'\subseteq[n-1]} \chi_{S'}(\bar{x}') c(S' \cup \{n\}). \tag{S16}$$

Let us focus now on the term $c(S' \cup \{n\})$. According to Eq. (S13), $c(S' \cup \{n\})$ can be computed as a sum of cumulants over all possible set partitions of $S' \cup \{n\}$. An arbitrary partition, π, of this set will have the form $\pi = \pi' \cup \{R \cup \{n\}\}$, where $R \subseteq S'$, and π' is a partition of $S' \setminus R$. We can see this by noticing that any partition of $S' \cup \{n\}$ necessarily has a block that contains n. This block can contain other elements of S', so it will generally have the form $\{R \cup \{n\}\}$ with $R \subseteq S'$. Since all the blocks in the partition must be mutually disjoint, all the blocks that are different from $\{R \cup \{n\}\}$ must not contain any of the elements in R. Thus they will form a collection of non-empty, mutually disjoint subsets of $S' \setminus R$ whose union is precisely $S' \setminus R$, that is, a partition of $S' \setminus R$. With this result in mind, we can see that

$$\sum_{\pi \in \mathcal{P}(S' \cup \{n\})} \prod_{b \in \pi} \equiv \sum_{R \subseteq S'} \sum_{\pi' \in \mathcal{P}(S' \setminus R)} \prod_{b \in \pi' \cup \{R \cup \{n\}\}} \tag{S17}$$

This allows us to write

$$\begin{aligned} c(S' \cup \{n\}) &= \sum_{R \subseteq S'} \sum_{\pi' \in \mathcal{P}(S' \setminus R)} \prod_{b \in \pi' \cup \{R \cup \{n\}\}} \kappa(b) \\ &= \sum_{R \subseteq S'} \kappa(R \cup \{n\}) \sum_{\pi' \in \mathcal{P}(S' \setminus R)} \prod_{b \in \pi'} \kappa(b). \end{aligned} \tag{S18}$$

Since $\sum_{\pi' \in \mathcal{P}(S' \setminus R)} \prod_{b \in \pi'} \kappa(b) = c(S' \setminus R)$, we obtain the following expression for $c(S' \cup \{n\})$:

$$c(S' \cup \{n\}) = \sum_{R \subseteq S'} \kappa(R \cup \{n\})\, c(S' \setminus R). \tag{S19}$$

Combining Eqs. (S16) and (S19), we may write

$$p(x_1, \ldots, x_n) = \frac{1}{2} p(x_1, \ldots, x_{n-1}) + \frac{(-1)^{x_n}}{2^n} \sum_{S' \subseteq [n-1]} \chi_{S'}(\bar{x}') \sum_{R \subseteq S'} \kappa(R \cup \{n\}) c(S' \setminus R). \tag{S20}$$

We can interchange the sums over $S' \subseteq [n-1]$ and $R \subseteq S'$ by noticing that these conditions imply that $R \subseteq [n-1]$, so we can sum over R instead of S' if we take into account that the final contribution of each R is obtained from all the $S' \subseteq [n-1]$ such that $R \subseteq S'$. In other words, we have that

$$\sum_{S' \subseteq [n-1]} \sum_{R \subseteq S'} \equiv \sum_{R \subseteq [n-1]} \sum_{\substack{S' \subseteq [n-1] \\ \text{s.t. } R \subseteq S'}} .$$

This allows us to write

$$\sum_{S' \subseteq [n-1]} \chi_{S'}(\bar{x}') \sum_{R \subseteq S'} \kappa(R \cup \{n\}) c(S' \setminus R) = \sum_{R \subseteq [n-1]} \kappa(R \cup \{n\}) \sum_{\substack{S' \subseteq [n-1] \\ \text{s.t. } R \subseteq S'}} \chi_{S'}(\bar{x}') c(S' \setminus R). \tag{S21}$$

Noticing that every subset $S' \subseteq [m-1]$ containing R can be written as $S' = T \cup R$, with $T \subseteq [n-1] \backslash R$, we can write

$$\sum_{\substack{S' \subseteq [n-1] \\ \text{s.t. } R \subseteq S'}} \chi_{S'}(\bar{x}') c(S' \setminus R) = \sum_{T \subseteq [n-1] \backslash R} \chi_{T \cup R}(\bar{x}') c(T). \tag{S22}$$

Eq. (S22) implies that Eq. (S20) will take the form

$$p(x_1, \ldots, x_n) = \frac{1}{2} p(x_1, \ldots, x_{n-1}) + \frac{1}{2^n} \sum_{R \subseteq [n-1]} \chi_{R \cup \{n\}}(\bar{x}_{R \cup \{n\}}) \kappa(R \cup \{n\}) \sum_{T \subseteq [n-1] \backslash R} \chi_T(\bar{x}_T) c(T), \tag{S23}$$

where $\bar{x}_A = (x_k \text{ for } k \in A)$, and we took into account that $(-1)^{x_n} \chi_{S'}(\bar{x}') = \chi_{R \cup \{n\}}(\bar{x}_{R \cup \{n\}}) \chi_T(\bar{x}_T)$. Introducing the marginal probability $p(\bar{x}_A)$, satisfying the relation

$$p(\bar{x}_A) = \frac{1}{2^{|A|}} \sum_{S \subseteq A} \chi_S(\bar{x}_A) c(S), \tag{S24}$$

we obtain

$$p(x_1, \ldots, x_n) = \frac{1}{2} p(x_1, \ldots, x_{n-1}) + \sum_{R \subseteq [n-1]} \frac{1}{2^{|R|+1}} \chi_{R \cup \{n\}}(\bar{x}_{R \cup \{n\}}) \kappa(R \cup \{n\})\, p(\bar{x}_{[n-1] \backslash R}). \tag{S25}$$

Using $p(x_1, \ldots, x_{n-1}) = p(x_1, \ldots, x_{n-1}, x_n) + p(x_1, \ldots, x_{n-1}, \bar{x_n})$ one can write the bias $\Delta_{x_n} = p(x_1, \ldots, x_{n-1}, x_n) - p(x_1, \ldots, x_{n-1}, \bar{x_n})$ as

$$\Delta_{x_n} = \sum_{R \subseteq [n-1]} \frac{1}{2^{|R|}} \chi_{R \cup \{n\}}(\bar{x}_{R \cup \{n\}}) \kappa(R \cup \{n\})\, p(\bar{x}_{[n-1] \backslash R}). \tag{S26}$$

This relation allows us to recursively compute the occurrence probability of a given bitstring using the cumulants of the distribution and previously computed marginals.

3 Approximating marginals efficiently with a chain rule

Before going into the details below, we need to clarify that in this section we will invert the order of variables in $p(x_1, x_2, \ldots, x_n)$ and instead use $p(x_n, x_{n-1}, \ldots, x_1)$, which is more natural for chain rule expressions related to conditional probabilities of the form $p(x_n | x_{n-1}, \ldots, x_1)$. In addition, we will first need a few auxiliary definitions:

- We will define the quantity $\gamma_A = \kappa(A) \chi(A)$, where $\chi(A) = (-1)^{\sum_{i \in A} x_i}$ is the parity function over the set of variables A and $\kappa(A)$ is its corresponding cumulant.

- We will need to use a compact notation for a particular form of sum over indices which are ordered alongside some other set of fixed indices which are not summed over. We will use

$$\sum_{\substack{(i_1>i_2>\ldots>i_k)\in[m,n-1]\setminus\{e_1,e_2,\ldots,e_\ell\} \\ (a_1,a_2,\ldots,a_{k+\ell})=\mathrm{ord}(i_1,i_2,\ldots,i_k,e_1,e_2,\ldots,e_\ell)}} \tag{S27}$$

  for a sum running over indices $i_1 > i_2 > \ldots > i_k$, ranging from m to $n-1$ apart from the elided indices $e_1, e_2, \ldots, e_\ell$, where we relabel the summed and elided indices together as $a_1, a_2, \ldots, a_{k+\ell}$ by rearranging them in decreasing order.

- We will use the expression $\binom{x}{d}_\Sigma$ as compact notation for

$$\binom{x}{d}_\Sigma = \sum_{i=0}^{d} \binom{x}{i} \approx \left(1 + \frac{d}{x}\right) \frac{x^d}{d!} \tag{S28}$$

  for the counting of cumulants up to order d. We will give details later on.

3.1 Double-elision method ($K = 5$)

The expansion of the probability $p(x_n, \bar{x})$ up to fifth order ($K = 5$) requires the use of three families of approximated marginals that are computed alongside $p(x_n, \ldots, x_1)$:

- Marginals $p^{(+)}(x_b, \ldots, x_a)$ containing all variables from x_a to x_b. We used the notation "(+)" to differentiate from the case where the variable with the lowest index is x_1, i.e. $p(x_n, \ldots, x_1)$.

- Marginals with all variables between x_1 and x_a except for one elision at x_e, i.e. $p^{(1)}(x_a, \ldots, \widetilde{x}_e, \ldots, x_1)$. We remind the reader that the tilde in $\widetilde{x}_e$ means that variable x_e is not present in the marginal (tracing out the variable).

- Marginals with all variables between x_1 and x_a except for two elisions at x_d and x_e. i.e. $p^{(2)}(x_a, \ldots, \widetilde{x}_e, \ldots, \widetilde{x}_d, \ldots, x_1)$.

The expansion of the probability $p(x_n, \ldots, x_1)$ up to fifth order ($K = 5$) then reads:

$$
\begin{aligned}
&p(x_n, \ldots, x_1) \\
&= \frac{1}{2}\big(1 + \gamma_{\{n\}}\big)\, p(x_{n-1}, \ldots, x_1) \\
&+ \frac{1}{4}\sum_{i=1}^{n-1} \gamma_{\{i,n\}} p^{(1)}(x_{n-1}, \ldots, \widetilde{x}_i, \ldots, x_1) \\
&+ \frac{1}{8}\sum_{i=2}^{n-1}\sum_{j=1}^{i-1} \gamma_{\{i,j,n\}} p^{(2)}\big(x_{n-1}, \ldots, \widetilde{x}_i, \ldots, \widetilde{x}_j, \ldots, x_1\big) \\
&+ \frac{1}{16}\sum_{i=3}^{n-1}\sum_{j=2}^{i-1}\sum_{k=1}^{j-1} \gamma_{\{i,j,k,n\}} p^{(+)}(x_{n-1}, \ldots, x_{i+1})\, p^{(2)}\big(x_{i-1}, \ldots, \widetilde{x}_j, \ldots, \widetilde{x}_k, \ldots, x_1\big), \\
&+ \frac{1}{32}\sum_{i=4}^{n-1}\sum_{j=3}^{i-1}\sum_{k=2}^{j-1}\sum_{\ell=1}^{k-1} \gamma_{\{i,j,k,\ell,n\}} p^{(+)}(x_{n-1}, \ldots, x_{i+1})\, p^{(+)}(x_{i-1}, \ldots, x_{j+1})\, p^{(2)}\big(x_{j-1}, \ldots, \widetilde{x}_k, \ldots, \widetilde{x}_\ell, \ldots, x_1\big),
\end{aligned}
\tag{S29}
$$

The approach is to replace the marginal $p(\bar{R})$, defined over the complement of the variables R, by an approximation. When the size of R is one or two we can use the marginals with one or two elisions, respectively, i.e. $p^{(1)}$ and $p^{(2)}$. Beyond this, we simplify the marginal by splitting it into a product of independent terms, such that they completely cover the set of variables R.

Our algorithm can be understood as a dynamical program, where we keep track of four tables, one for each type of approximated marginal $p^{(+)}(x_b, \ldots, x_a)$, $p^{(1)}(x_a, \ldots, \widetilde{x}_e, \ldots, x_1)$ and $p^{(2)}(x_a, \ldots, \widetilde{x}_e, \ldots, \widetilde{x}_d, \ldots, x_1)$, as well as the sampling probabilities $p(x_n, \bar{x})$. Each table is updated from a non-trivial combination of previously computed values.

At every step there is one term $p(x_n, \ldots, x_1)$ to compute consisting of a sum of size $\binom{n-1}{4}_\Sigma \approx O(n^4)$. The largest sum update is of order $O(M^4)$, and its total computing time $\sum_{i=1}^{M} \binom{i-1}{4}_\Sigma \approx O(M^5)$ operations, requiring only a memory storage of $O(M)$. This does not finalize the cost analysis, as we need to incorporate the cost of the approximated marginals that we detail below and we finalize the section with a summary of the total costs.

As we will see below a key aspect of the design of the approximated marginals is to ensure the cost of filling the tables corresponding to the approximated marginals do not degrade the asymptotic scaling of $O(n^4)$ for the nth time step and a final time asymptotic of $O(M^5)$. This is achieved at the cost of storing $O(M^3)$ tables data during the dynamical program for each concurrent thread

alongside the storage of the $\binom{M}{5} \approx O(M^5)$ global cumulants of order up to $K = 5$.

3.1.1 Marginal with non-fixed last variable

The approximated marginals $p^{(+)}(x_n, \ldots, x_\ell)$ containing all variables between x_n and x_ℓ being present are approximated by the following update rule:

$$\begin{aligned}
&p^{(+)}(x_n, \ldots, x_\ell) \\
&= \frac{1}{2}\left(1 + \gamma_{\{n\}}\right) p^{(+)}(x_{n-1}, \ldots, x_\ell) \\
&+ \frac{1}{4}\sum_{i=\ell}^{n-1} \gamma_{\{i,n\}} p^{(+)}(x_{n-1}, \ldots, x_{i+1})\, p^{(+)}(x_{i-1}, \ldots, x_\ell) \\
&+ \frac{1}{8}\sum_{i=\ell+1}^{n-1}\sum_{j=\ell}^{i-1} \gamma_{\{i,j,n\}} p^{(+)}(x_{n-1}, \ldots, x_{i+1})\, p^{(+)}\left(x_{i-1}, \ldots, x_{j+1}\right) p^{(+)}\left(x_{j-1}, \ldots, x_\ell\right)
\end{aligned} \tag{S30}$$

At every step of the chain rule (nth step), there are $n-1$ terms $p^{(+)}(x_n, \ldots, x_\ell)$ to compute, as $\ell \in [1, n-1]$:

- Each term consists of a sum of size $\binom{n-1}{2}_\Sigma \approx n^2/2$.
- A total of at most $5(n-1)\binom{n-1}{2}_\Sigma \approx O(n^3)$ computing operations.

In the process of generating a sample of size M we will need:

- A table of total size $\binom{M}{2}$ terms, resulting from having two free parameters (n, ℓ) ranging from 1 to M.
- The longest update has a time complexity of $(M-1)\binom{M-1}{2}_\Sigma \approx O(M^3)$.
- The total computing time is $\sum_{i=1}^{M}(i-1)\binom{i-1}{2}_\Sigma \approx O(M^4)$ operations.

3.1.2 Marginal with one elided variable

The approximated marginals $p^{(1)}(x_n, \ldots, \widetilde{x}_e, \ldots, x_1)$ with all variables between x_n and x_1 being present except for the elided variable x_e with index $e \in [1, n-1]$ can be computed from the following update rule:

$$
\begin{aligned}
&p^{(1)}(x_n, \dots, \widetilde{x}_e, \dots, x_1) \\
&= \frac{1}{2}\left(1 + \gamma_{\{n\}}\right) p^{(1)}(x_{n-1}, \dots, \widetilde{x}_e, \dots, x_1) \\
&+ \frac{1}{4} \sum_{\substack{i \in [1,n-1] \setminus \{e\} \\ (a,b) = \mathrm{ord}(i,e)}} \gamma_{\{i,n\}} p^{(2)}(x_{n-1}, \dots, \widetilde{x}_a, \dots, \widetilde{x}_b, \dots, x_1) \\
&+ \frac{1}{8} \sum_{\substack{(i>j) \in [1,n-1] \setminus \{e\} \\ (a,b,c) = \mathrm{ord}(i,j,e)}} \gamma_{\{i,j,n\}} p^{(+)}(x_{n-1}, \dots, x_{a+1})\, p^{(2)}(x_{a-1}, \dots, \widetilde{x}_b, \dots, \widetilde{x}_c, \dots, x_1)
\end{aligned} \tag{S31}
$$

At every step there are $n-1$ terms $p^{(1)}(x_{n-1}, \dots, \widetilde{x}_e, \dots, x_1)$ to compute, one for each value of the elided variable $e \in 1, n-1$:

- Each term consists of a sum of size $\binom{n-2}{2}_\Sigma \approx n^2/2$.
- A total of at most $4(n-1)\binom{n-2}{2}_\Sigma \approx O(n^3)$ computing operations.

In the process of generating a sample of size M we will need:

- A tableau of total size $\binom{M}{2}$ terms
- The longest update takes a time $(M-1)\binom{M-2}{2}_\Sigma \approx O(M^3)$
- The total computing time $\sum_{i=2}^{M}(i-1)\binom{i-2}{2}_\Sigma \approx O(M^4)$ operations

3.1.3 Marginal with two elided variables

The approximated marginals $p^{(2)}(x_{n-1}, \dots, \widetilde{x}_e, \dots, \widetilde{x}_d, \dots, x_1)$ with all variables between x_n and x_1 being present except for the two elided variables x_d and x_e with indices in $[1, n-1]$ can be computed from the following update rule:

$$
\begin{aligned}
&p^{(2)}(x_n, \dots, \widetilde{x}_e, \dots, \widetilde{x}_d, \dots, x_1) \\
&= \frac{1}{2}\left(1 + \gamma_{\{n\}}\right) p^{(2)}(x_{n-1}, \dots, \widetilde{x}_e, \dots, \widetilde{x}_d, \dots, x_1) \\
&+ \frac{1}{4} \sum_{\substack{i \in [1,n-1] \setminus \{e,d\} \\ (a,b,c) = \mathrm{ord}(i,e,d)}} \gamma_{\{i,n\}} p^{(+)}(x_{n-1}, \dots, x_{a+1})\, p^{(2)}(x_{a-1}, \dots, \widetilde{x}_b, \dots, \widetilde{x}_c, \dots, x_1)
\end{aligned} \tag{S32}
$$

At every step there are $\binom{n-1}{2}$ terms $p^{(2)}(x_n, \ldots, \widetilde{x}_e, \ldots, \widetilde{x}_d, \ldots, x_1)$ to compute, as we have two elided variables $\{e, d\} \in [1, n-1]$:

- Each term consists of a sum of size $n-3$.
- A total of $3\binom{n-1}{2}(n-3) \approx O(n^3)$ computing operations.

In the process of generating a sample of size M we will need:

- a tableau of total size $\binom{M}{3}$ terms
- the longest update takes a time $\binom{M-1}{2}(M-3) \approx O(M^3)$
- the total computing time $\sum_{i=3}^{M} \binom{i-1}{2}(i-3) \approx O(M^4)$ operations

3.1.4 Total cost estimates

In the following table we summarize the information presented above related to the cost of storing and computing the approximated marginals $p^{(+)}$, $p^{(1)}$, $p^{(2)}$ and the sampling distribution p for an nth step of the chain rule and the generation of a single sample of size M. Finally, we also give the order on the expansion in terms of cumulants for each one.

In order to retain a time scaling of $O(M^5)$, we needed to truncate the cumulant expansions by at least one order for $p^{(+)}$ and $p^{(1)}$, and at least two orders for $p^{(2)}$ as there is a factor of $O(M)$ more values to compute. In practice, for the Jiuzhang experiments, we found that truncating the expansions for $p^{(+)}$, $p^{(1)}$ and $p^{(2)}$ by an additional order barely degraded the quality of the generated samples, which is why the total time complexity for computing each of the respective tables is $O(M^4)$ rather than $O(M^5)$. This choice also dramatically improves the sample generation rate with our implementation.

4 Correlators and cumulants of GBS with binary outcomes

Before we derive the correlators of GBS with binary outcomes, we will do as a warm-up the qubit case with the density matrix $\hat{\varrho}$. It is easy to see that the action of the $\hat{Z}$ gate on the computational

Aux. Marg.	$p^{(+)}$	$p^{(1)}$	$p^{(2)}$	p
Per step:				
Terms	$O(n)$	$O(n)$	$O(n^2)$	1
Time per term	$O(n^2)$	$O(n^2)$	$O(n)$	$O(n^4)$
Time	$O(n^3)$	$O(n^3)$	$O(n^3)$	-
Per sample:				
Memory	$O(M^2)$	$O(M^2)$	$O(M^3)$	$O(M)$
Longest update	$O(M^3)$	$O(M^3)$	$O(M^3)$	$O(M^4)$
Total time	$O(M^4)$	$O(M^4)$	$O(M^4)$	$O(M^5)$
Exp. Order	3	3	2	5

Table S1: **Auxiliary marginal cost summary.** Computational costs of the dynamic programming tables used in the $K = 5$ double-elision algorithm. The columns correspond to the auxiliary marginals with non-fixed last variable $p^{(+)}$, marginals with one and two elided variables $p^{(1)}$ and $p^{(2)}$, and the sampling probabilities p. The upper block gives the number of terms and update cost at the nth step of the chain rule, while the lower block gives the memory requirement, longest update, and total runtime for generating one sample of size M. The final row gives the cumulant expansion order retained for each table, chosen so that the auxiliary marginal updates do not increase the overall $O(M^5)$ sampling-time scaling.

basis, i.e. $\hat{Z}\left|x\right\rangle = (-1)^x \left|x\right\rangle$ is characterized by the single-bit parity function. One can generalize this and define the operator $\hat{Z}_A$ such that $\hat{Z}_A \left|\bar{x}\right\rangle = (-1)^{\sum_{k\in A} x_k} \left|\bar{x}\right\rangle$, where

$$\hat{Z}_A = \left(\bigotimes_{i\in A} \hat{Z}_i\right) \otimes \hat{\mathbb{I}}_{\bar{A}}, \tag{S33}$$

and $\hat{\mathbb{I}}_{\bar{A}}$ is the identity operator over the complement set to A. It is easy to see that

$$\mathrm{Tr}\left[\hat{Z}_A \hat{\varrho}\right] = \mathrm{Tr}\left[\hat{\varrho}_A \bigotimes_{i\in A} \hat{Z}_i\right] = \frac{1}{2^{|A|}} \sum_{\bar{x}\in\{0,1\}^{|A|}} p(\bar{x}_A)(-1)^{\sum_{k\in A} x_k}, \tag{S34}$$

where $p(\bar{x}_A)$ is the probability of outcome $\bar{x}_A$ if we measure $\hat{\varrho}_A$ in the computational basis, i.e. the correlator corresponding to the probability distribution resulting from measuring the quantum state $\hat{\varrho}$ into the computational basis.

4.1 Correlators of GBS with binary outcomes

The outcome of a threshold detector measurement of a multimode Gaussian state, $\hat{\varrho}$, is a bitstring $\bar{x} = (x_1, \ldots, x_M)$ with M the number of modes in the system. The outcome $x_k = 0$ indicates that no photons have been detected at mode k, while $x_k = 1$ indicates that one or more photons arrived at the detector (i.e. that the detector "clicked"). For each mode, this measurement is represented by the two-element POVM $\{|0_k\rangle\langle 0_k|, \hat{\mathbb{I}}_k - |0_k\rangle\langle 0_k|\}$, where $\hat{\mathbb{I}}_k$ is the identity operator at mode k and $|0_k\rangle$ the vacuum state in the same mode. Similarly to qubits, we have $Z = 2|0\rangle\langle 0| - \hat{\mathbb{I}}$, one can define a generalized version of the Z operator to the Fock basis as $Z = 2|0_k\rangle\langle 0_k| - \hat{\mathbb{I}}_k$, which after some derivation shown below, leads to

$$c(S) = \mathrm{Tr}\left[\hat{\varrho}\left(\bigotimes_{k\in S}[2|0_k\rangle\langle 0_k| - \hat{\mathbb{I}}_k]\right)\otimes \hat{\mathbb{I}}_{[M]\setminus S}\right], \tag{S35}$$

which can be further developed as a sum of Gaussian integrals as we detail below.

4.1.1 Formal derivation

Given a multimode measurement outcome $\bar{x}$, let C be the subset of $[M] = \{1, \ldots, M\}$ for which $x_k = 1$ for all $k \in C$. Then, we can associate $\bar{x}$ to the measurement of the operator

$$\hat{\Pi}(\bar{x}) = \bigotimes_{k\in C}(\hat{\mathbb{I}}_k - |0_k\rangle\langle 0_k|) \bigotimes_{j\in[M]\setminus C} |0_k\rangle\langle 0_k|. \tag{S36}$$

Alternatively, we can write this operator as

$$\hat{\Pi}(\bar{x}) = \bigotimes_{k=1}^{M}(\hat{\mathbb{I}}_k - |0_k\rangle\langle 0_k|)^{x_k}(|0_k\rangle\langle 0_k|)^{1-x_k}. \tag{S37}$$

From these relations, one can readily see that $\sum_{\bar{x}\in\{0,1\}^M} \hat{\Pi}(\bar{x}) = \hat{\mathbb{I}}$, with $\hat{\mathbb{I}}$ the identity operator over the M-mode Hilbert space of the system.

The probability of obtaining the outcome $\bar{x}$ when measuring the system in the state $\hat{\varrho}$ is given by

$$p(\bar{x}) = \mathrm{Tr}[\hat{\Pi}(\bar{x})\hat{\varrho}]. \tag{S38}$$

When combined with the definition of correlator in Eq. (S8) , we can use this expression to associate a Hermitian operator to each correlator $c(S)$. Indeed, using the notation $\hat{\mathbb{I}}_A$ for the identity operator

over the Hilbert space associated to the modes in A, we have

$$\begin{aligned} c(S) &= \sum_{\bar{x}\in\{0,1\}^M} p(\bar{x}) \left(\prod_{k\in S}(-1)^{x_k}\right) \\ &= \mathrm{Tr}\left[\hat{\varrho} \sum_{\bar{x}\in\{0,1\}^M} \left(\prod_{k\in S}(-1)^{x_k}\right)\left(\bigotimes_{\ell=1}^{M}(\hat{\mathbb{I}}_\ell - |0_\ell\rangle\langle 0_\ell|)^{x_\ell}(|0_\ell\rangle\langle 0_\ell|)^{1-x_\ell}\right)\right] \\ &= \mathrm{Tr}\left[\hat{\varrho} \bigotimes_{\ell=1}^{M} \sum_{x_\ell\in\{0,1\}} \left((-1)^{x_{\ell\in S}}(\hat{\mathbb{I}}_\ell - |0_\ell\rangle\langle 0_\ell|)^{x_\ell}(|0_\ell\rangle\langle 0_\ell|)^{1-x_\ell}\right)\right] \end{aligned} \tag{S39}$$

where $(-1)^{x_{\ell\in S}} = (-1)^{x_\ell}$ if $\ell \in S$ or 1 otherwise. The sum over both values of x_ℓ contributes a term $\hat{\mathbb{I}}_\ell$ when $\ell \notin S$ and $2|0_\ell\rangle\langle 0_\ell| - \hat{\mathbb{I}}_\ell$ when $\ell \in S$, leading to

$$c(S) = \mathrm{Tr}\left[\hat{\varrho}\left(\bigotimes_{k\in S}[2|0_k\rangle\langle 0_k| - \hat{\mathbb{I}}_k]\right)\otimes \hat{\mathbb{I}}_{[M]\setminus S}\right], \tag{S40}$$

Note that the operators $\{2|0_k\rangle\langle 0_k| - \hat{\mathbb{I}}_k, \hat{\mathbb{I}}_k\}_{k=1}^{M}$ do not form a POVM.

Taking into account that

$$\bigotimes_{k\in S}[2|0_k\rangle\langle 0_k| - \hat{\mathbb{I}}_k] = \sum_{R\subseteq S}\left[\bigotimes_{k\in R}(2|0_k\rangle\langle 0_k|)\bigotimes_{\ell\in S\setminus R}(-\hat{\mathbb{I}}_\ell)\right], \tag{S41}$$

we can recast $c(S)$ as

$$c(S) = (-1)^{|S|}\sum_{R\subseteq S}(-2)^{|R|}\langle 0_R|\hat{\varrho}_R|0_R\rangle, \tag{S42}$$

where $|0_R\rangle = \bigotimes_{k\in R}|0_k\rangle$, and $\hat{\varrho}_R$ is the reduced state obtained by tracing out all the modes that are not in R, i.e. $\hat{\varrho}_R = \mathrm{Tr}_{[M]\setminus R}(\hat{\varrho})$.

The Gaussian state $\hat{\varrho}$ can be completely parametrized by a $2M \times 2M$ covariance matrix $\boldsymbol{\sigma}$ and a vector of first moments $\boldsymbol{\mu}$ of length $2M$ (*40*). The reduced state $\hat{\varrho}_R$ is also Gaussian, and can be parametrized by a $2|R| \times 2|R|$ covariance matrix σ_R and a vector of means $\boldsymbol{\mu}_R$, which can be obtained from $\boldsymbol{\sigma}$ and $\boldsymbol{\mu}$ according to the following prescription: $\boldsymbol{\mu}_R$ is obtained by removing the kth and $(k+M)$th elements of $\boldsymbol{\mu}$ for $k \in [M] \setminus R$, while $\boldsymbol{\sigma}_R$ is obtained by removing the kth and $(k+M)$th rows and columns of $\boldsymbol{\sigma}$ for $k \in [M] \setminus R$ (*40*).

In terms of $\boldsymbol{\sigma}_R$ and $\boldsymbol{\mu}_R$, it can be shown that (*41, 42*)

$$\langle 0_R|\hat{\varrho}_R|0_R\rangle = \frac{\exp\left[-\frac{1}{2}\boldsymbol{\mu}_R^{\mathrm{T}}\left(\boldsymbol{\sigma}_R + \frac{\hbar}{2}\mathbb{I}\right)^{-1}\boldsymbol{\mu}_R\right]}{\sqrt{\det\left[\frac{1}{\hbar}\left(\boldsymbol{\sigma}_R + \frac{\hbar}{2}\mathbb{I}\right)\right]}}, \tag{S43}$$

with $\mathbb{I}$ the $2|R|\times 2|R|$ identity matrix. For the non-displaced ($\boldsymbol{\mu} = 0$) case of the Jiuzhang experiments, the correlators $c(S)$ take the following form as a function of $\boldsymbol{\sigma}$:

$$c(S) = (-1)^{|S|}\sum_{R\subseteq S}\frac{(-2)^{|R|}}{\sqrt{\det\left[\frac{1}{\hbar}\left(\boldsymbol{\sigma}_R + \frac{\hbar}{2}\mathbb{I}\right)\right]}}. \tag{S44}$$

4.2 Cumulant expression

The cumulants κ required in the sampling procedure are computed from the correlators $c(S)$ for all sets S of size 1 to K composed of elements of $[M]$ using the relation

$$\kappa(S) = \sum_{\pi\in\mathcal{P}(S)}(-1)^{|\pi|-1}(|\pi|-1)!\prod_{B\in\pi}c(B). \tag{S45}$$

5 Comparison to other implementations and experiment

Here we discuss the performance and scalability of the two main algorithms for simulating GBS, the first one presented in 2022 in Ref. (*22*) (which we shall refer to as 'the Google methods'), the second presented in 2024 in Ref. (*18*) (which we shall refer to as 'the Chicago method') for which we provide a direct comparison for J2-P65-5 and J3-high experiments.

5.1 Google methods

Ref. (*22*) details two alternative algorithms to approximate experimental GBS.

5.1.1 Boltzmann machine

The first approach consists of training a Boltzmann machine of distribution

$$p(\mathbf{z}) = \frac{1}{Z}\exp\left(\sum_a \lambda_a z_a + \sum_{a<b}\lambda_{a,b}z_a z_b + \sum_{a<b<c}\lambda_{a,b,c}z_a z_b z_c + \dots\right) \tag{S46}$$

including all first k summands using gradient descent of the log-likelihood. This requires estimating the correlations of the GBS of the ground truth and of the Boltzmann machine. If the first can be computed exactly up to machine precision up, computing a Torontonian with scaling $O(M^3 2^K)$ equivalent to the computing cost of our correlators computations, the Boltzmann machine correlations are estimated through sampling. The required number of samples being inversely proportional to the required precision in marginal probabilities combined with the exponential decay of the correlations with k makes the training cost scale exponentially with k. To alleviate this cost Ref. (*22*) trained a $k = 2$ Boltzmann machine using a TAP mean-field approximation, which gives direct mapping between the GBS correlations and the BM parameters up to order 2. The sampling algorithm runs in time $O(M^2)$, per sample.

The Google team carried out a series of comparisons on the approximation algorithm against the experimental data to approximate the ground truth. They used as benchmarks: (i) Pearson correlations; (ii) total click distributions; (iii) total variation distance of marginals up to 15 modes; (iv) KL-divergence of marginals up to 15 modes; (v) XEB of photon sectors. Except for (iii) and (iv) the remaining tests are the same as those we implement in our work. The $k = 2$-TAP Boltzmann machine achieved mixed results when compared against experiments. If it outperforms the J1 experiment on all test except the Pearson correlations, for J2 it only succeeds for high-brightness experiments. More importantly, it is easy to discriminate this emulator from the experiment by using the Pearson correlation test for orders $k \leq 3$.

5.1.2 Greedy heuristic

Alternatively, Ref. (*22*) proposes to generate L samples of size M, by populating a matrix $L \times M$, where iteratively a Greedy algorithm updates the columns in order to set them as close as possible to theoretical marginals. The algorithm runs in time $O(M^K 2^K L)$, where K is the highest order of the marginals that we try to match. Ref. (*22*) shows that the Greedy $k = 3$ outperforms the experiment on all metrics except the Pearson correlation test for orders $k = 4$ and $k = 5$ (for $k \geq 6$ experimental data is indistinguishable from zero).

Even if increasing the order k of the Greedy algorithm could potentially match the experiment for higher order k, the algorithm has some disadvantageous features with respect to our proposed emulator. Firstly the scaling $O(M^K 2^K L)$ is more disadvantageous, as our algorithm has $O(M^K 2^K)$

for training and $O(M^K L)$ for sampling, saving a significant factor L in the training and 2^K in the sampling. More importantly, the Greedy algorithm does not produce i.i.d. samples, an intrinsic property to our samples, and fails to reproduce probabilities of $O(1/L)$. This last factor makes the quality of the samples vary with the number of samples created, which can be exploited to build a statistical test detecting this particular spoofing strategy in a sample batch.

5.2 The Chicago method

There are two main stages of the algorithm: formation of the matrix product state (MPS) which is costly, but need only be done once, and sampling, which is less expensive but must of course be repeated every time we generate a sample. The MPS represents the output state of some quantum circuit under the influence of photon loss. It is by taking the effect of photon loss into account that the Chicago method achieves advantage over prior algorithms. The idea behind the approach is that losses allow the output state to be written as a convex mixture of Gaussian states with significantly lower squeezing, which can be efficiently created, stored, and sampled from, however, a random set of local displacement operations need to be applied when generating each sample. Local operations have no major impact on the scalability of sampling from an MPS, the costs are mostly equivalent to those of the pure Gaussian state with weaker squeezing resulting from the convex decomposition facilitated by the presence of losses. The prepared MPS is saved to disk, and then loaded separately for the sampling calculation.

5.2.1 Time complexity

MPS Formation First we consider the asymptotic time complexity of the MPS formation stage of the Chicago method. This is reported as $O(cMd\chi^2)$ where χ is the bond dimension of the MPS tensors, d is the physical dimension of the local Hilbert space, i.e. the maximum number of photons per mode, M is the number of modes, and c is "a parameter that depends on the system's characteristics". The bond dimension χ is a parameter of the MPS representation itself that determines the available degrees of freedom – the ability to compress the representation of a state by limiting χ is at the core of tensor network methods. Crucially, for quantum states, the bond dimension is linked to the Schmidt rank of any bipartition of the system and therefore limits

the amount of entanglement that can be represented by the MPS. This means that MPS (and other tensor decompositions) can be used to form a well controlled approximation of a quantum state, and the lower the entanglement in the system, the more efficiently the state can be represented. They use values of χ ranging from 2 to 10,000, with $\chi = 10,000$ for the majority of their numerical simulations. The physical dimension d is simpler to understand, being the maximum number of photons that may appear in any given mode. It obviously must be truncated to some finite value, $d = 4$ was used during MPS construction, while $d = 10$ during sampling to allow for the contribution of additional photons resulting from the displacement operation. The number of modes M is a parameter of the experiment being simulated, and ranges from 72 to 288 in the numerical results. The final parameter, c, is the complexity of computing a hafnian, which is exponential in the mean number of photons of each mode's thermal state, and consequently dominated by the largest such photon number in the system. More precisely, the scaling is given in the supplementary material of Ref. (*18*) as $\widetilde{O}(2^{(\mathbf{n}_1+\mathbf{n}_2)/2})$, where $\mathbf{n}_i$ are photon occupation patterns appearing in expectation values $\langle n_2|U|n_1\rangle$ leading to the tensor terms. Due to the low squeezing resulting from the convex decomposition, these occupation patterns have limited total number of photons, making the hafnian calculation more efficient than directly sampling from the output state using the best-known classical algorithm.

Sampling The serial complexity of the sampling procedure is dominated by the bond dimension of the MPS tensors, with an asymptotic complexity of $O(M\chi^2 d)$ *per sample*. Again, we note that during the sampling phase the parameter d, the physical dimension of the MPS tensor which limits the number of photons allowed per site, is increased from 4 to 10. This is to accommodate an operation in which photons in the prepared state are randomly displaced immediately before each sample is taken, and which may cause the photon number to increase on particular sites. In practical terms there is more work to do in the sampling procedure than the MPS preparation procedure whenever the number of samples N is greater than c the complexity of computing a hafnian. This is reflected in the runtimes for each stage, where constructing the MPS took around 10 minutes for the largest size problems, while generating ten million samples took around an hour, despite the parallelism available during the sampling step.

5.2.2 Space complexity

The dominant data structure is the matrix product state which has a total size of $O(Md\chi^2)$, with one tensor per mode – once again the bond dimension, χ, is dominant. This structure is at its largest during the sampling procedure when d is increased to 10. Since each element of the tensor is a 16 byte complex double, this means each tensor requires 16 GB of memory at $\chi = 10,000$. The GPUs used in this experiment had 40 GiB of memory each, so were only capable of carrying two site tensors at once, and at least one copy is required during the sample calculation.

5.2.3 Parallel scalability

Due to the large size of the MPS tensors the principle bottleneck in scaling the code is the available memory on each GPU. In both the MPS preparation procedure, and the sampling procedure, only one site tensor is loaded per GPU meaning for a system with M modes, exactly M GPUs are used. Both stages do take advantage of the shared memory thread-parallelism available on each GPU, with multiple hafnians calculated in parallel during the MPS construction phase, and multiple samples calculated in parallel during the sampling phase. This parallelism is limited in both cases by the memory available on the GPU – in the sampling stage for the largest system the authors fix the number of samples calculated in parallel to 100.

The MPS construction phase benefits from requiring no communication between GPUs, whereas the sampling stage essentially requires a full contraction of the network so vectors of length χ must be passed from GPU to GPU to calculate the overall sample result. This step can however be pipelined, with a number of steps equal to $M + \frac{N}{B} - 1$ where M is the number of modes and thus GPUs and pipeline stages, N is the total number of samples being calculated, and B is the number of samples calculated in parallel on each GPU so there are M timesteps before any result is produced, and then $\frac{N}{B}$ results produced at each subsequent timestep. The complexity of each step in the pipeline is, as described above, $O(\chi^2 d)$.

Due to the limitation of one GPU per mode, there is no scalability in terms of number of GPUs, but runtime would nevertheless be improved by GPUs with more memory, which would allow more hafnians to be calculated in parallel giving a linear improvement to the MPS construction procedure, or more samples to be calculated in parallel (B) on each GPU in the sampling stage.

The biggest disadvantage in scaling the simulation to larger GBS experiments is the need for one GPU per mode, making the simulation of few thousand modes, like in the recent Jiuzhang 4.0 (*7*) experiment, very costly to implement.

5.2.4 Comparison to our simulator

To conclude this section we present a comparison between the performance of the double-elision sampler and that of the Chicago algorithm (a link to samples obtained using this algorithm can be found in (*18*)). We focus on the two brightest configurations considered in the main text, i.e., the J2-P65-5 and J3-high experiments. On this account, we only show a comparison of click cumulants (Fig. S1), and their corresponding Pearson correlation coefficients (Fig. S2). In both of these figures we can see that the Chicago algorithm slightly outperforms the double-elision sampler. Note however that this does not undermine the capacity of the double-elision sampler of outperforming, with far less computational resources than the Chicago algorithm, the J2-P65-5 and J3-high experiments.

6 Benchmarking theory and implementation

In this section, we explain the different benchmarking tools used in the validation of our sampler. For all these tests, we compared sample metrics with those predicted by the ground truth of the Jiuzhang experiments, which in turn can be obtained using the data that has been made publicly available by the authors of the experiments (*43–45*). Before explaining the theory and purpose of the different validation methods, in the following, we will explain how to obtain the ground truth that each of these tests uses.

The Jiuzhang 2.0 experiment consists of a set of $k = 25$ two-mode squeezed states with different squeezing parameters that are sent into a lossy linear interferometer with $M = 144$ output ports. The light exiting the interferometer is measured using threshold detectors. The authors of the experiment made available a list of squeezing parameters $\boldsymbol{r} = (r_1, \ldots, r_k)$ and a $2k \times M$ complex transmission matrix $\boldsymbol{T}$ representing the action of the interferometer. The ground truth distribution of the experiment corresponds to the probability distribution of the threshold detection outcomes, $p(\bar{x})$, that are obtained after the measurement of the Gaussian state exiting the interferometer. This

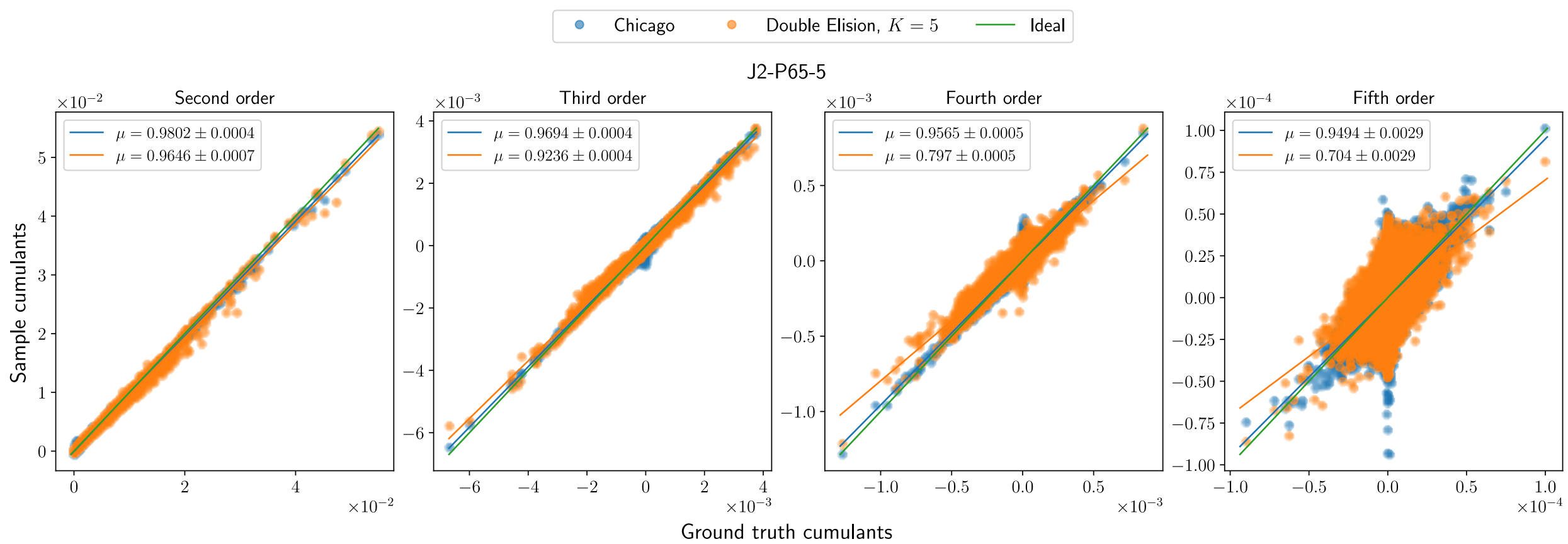

(a)

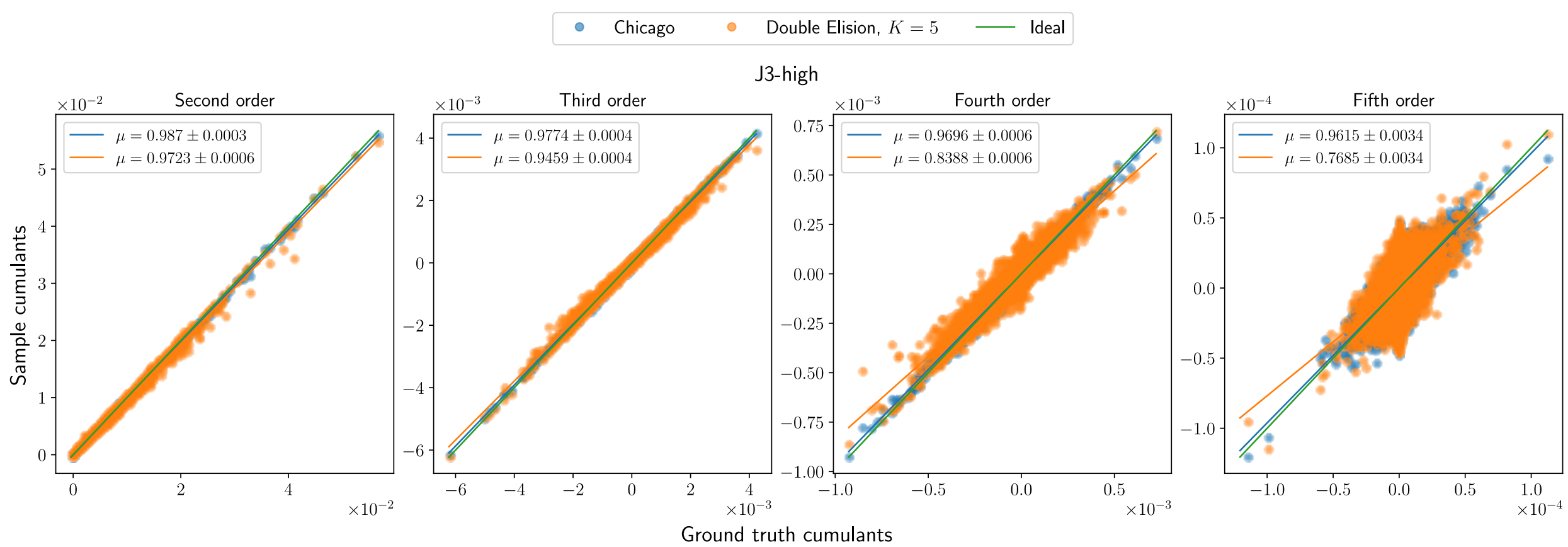

(b)

Figure S1: **Comparison of click cumulants for Chicago and double-elision samples.** Comparison between the estimated click cumulants (orders 2 to 5), from Chicago (blue circles) and double-elision samples with $K = 5$ (orange circles), and those predicted by the ground truth of the experiment (green line) for J2-P65-5 (a) and J3-high (b). The vertical axis corresponds to cumulants obtained from samples, while the horizontal axis corresponds to the ground truth (i.e., theoretical) cumulants. The blue and orange lines represent the linear fit between sample cumulants and those from the ground truth. The slope of each linear fit is shown in the legends. All cumulants were estimated using 10^7 samples.

distribution is completely parametrized by the quadrature covariance matrix of the output state, $\boldsymbol{\sigma}_{\mathrm{GT}}$, which can be obtained from $\boldsymbol{r}$ and $\boldsymbol{T}$ as follows.

The k input two-mode squeezed states form a $2k$-mode non-displaced Gaussian state that is

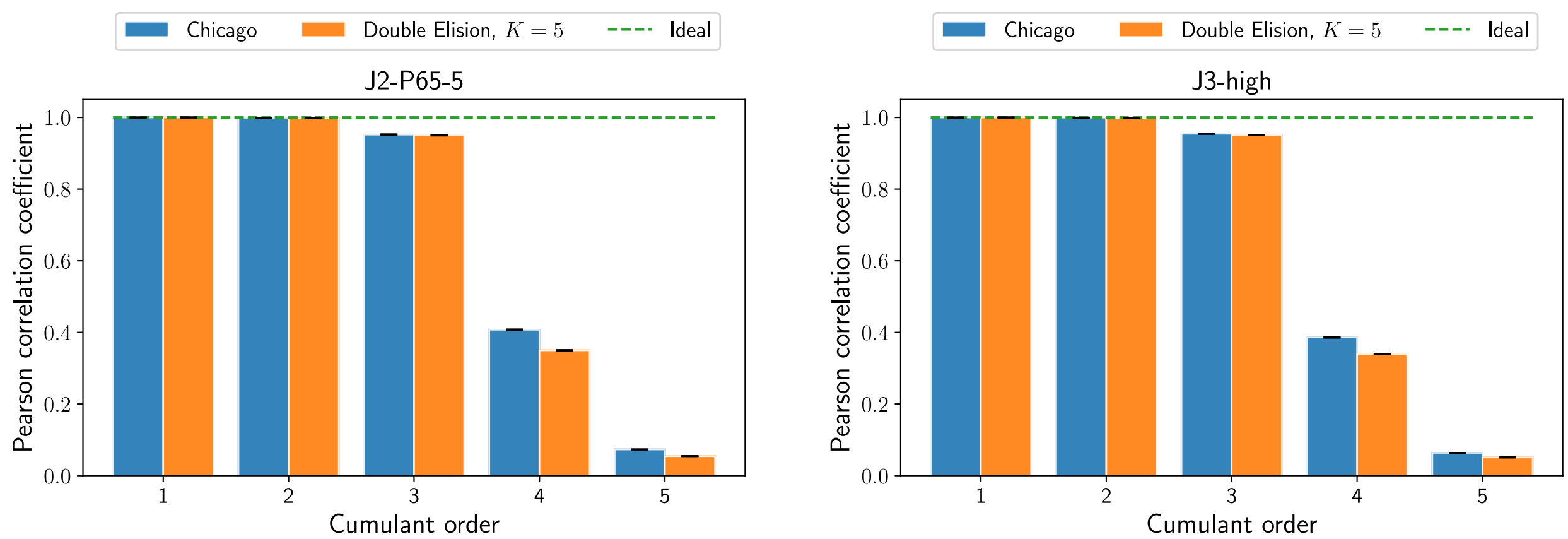

(a) (b)

Figure S2: **Comparison of Pearson correlation coefficients for Chicago and double-elision samples.** Pearson correlation coefficients between Chicago and double-elision ($K = 5$) sample and ground truth cumulants for the J2-P65-5 (a) and J3-high experiments. The green dashed line indicates the ideal value of these coefficients, which is equal to 1. Error bars were obtained using 100 bootstrapping resamples.

completely parametrized by a $4k \times 4k$ covariance matrix

$$\boldsymbol{\sigma}_{\text{IN}} = \frac{\hbar}{2} \left[\bigoplus_{j=1}^{k} \begin{pmatrix} \cosh(r_j) & \sinh(r_j) \\ \sinh(r_j) & \cosh(r_j) \end{pmatrix} \right] \oplus \left[\bigoplus_{j=1}^{k} \begin{pmatrix} \cosh(r_j) & -\sinh(r_j) \\ -\sinh(r_j) & \cosh(r_j) \end{pmatrix} \right]. \tag{S47}$$

By defining

$$\boldsymbol{V} = \begin{pmatrix} \text{Re}(\boldsymbol{T}) & -\text{Im}(\boldsymbol{T}) \\ \text{Im}(\boldsymbol{T}) & \text{Re}(\boldsymbol{T}) \end{pmatrix}, \tag{S48}$$

the $2M \times 2M$ ground truth covariance matrix can be computed as

$$\boldsymbol{\sigma}_{\text{GT}} = \boldsymbol{V} \boldsymbol{\sigma}_{\text{IN}} \boldsymbol{V}^{\text{T}} + \frac{\hbar}{2} \left(\mathbb{I} - \boldsymbol{V} \boldsymbol{V}^{\text{T}} \right). \tag{S49}$$

Given a measurement outcome $\bar{x}$, the corresponding ground truth probability is computed as

$$p(\bar{x}) = \frac{\text{tor}[\boldsymbol{O}_{(\bar{x})}]}{\sqrt{\det(\boldsymbol{\Sigma}_{\text{GT}})}}, \tag{S50}$$

where $\boldsymbol{O} = \mathbb{I} - \boldsymbol{\Sigma}_{\text{GT}}^{-1}$, $\boldsymbol{\Sigma}_{\text{GT}} = \frac{1}{2}\mathbb{I} + \frac{1}{\hbar} \boldsymbol{R} \boldsymbol{\sigma}_{\text{GT}} \boldsymbol{R}^{\dagger}$, and $\boldsymbol{R} = \frac{1}{\sqrt{2}} \left(\begin{smallmatrix} \mathbb{I} & i\mathbb{I} \\ \mathbb{I} & -i\mathbb{I} \end{smallmatrix} \right)$. The matrix $\boldsymbol{O}_{(\bar{x})}$ is obtained by removing the kth and $(k + M)$th rows and columns from $\boldsymbol{O}$ whenever $x_k = 0$. Finally, $\text{tor}(\boldsymbol{M})$ is the

"Torontonian" (*28*) of the $2n \times 2n$ matrix $\boldsymbol{M}$, and it is computed as

$$\mathrm{tor}(\boldsymbol{M}) = \sum_{R\subseteq[n]} \frac{(-1)^{|R|}}{\sqrt{\det(\mathbb{I} - \boldsymbol{M}_R)}}, \tag{S51}$$

where, again, $\boldsymbol{M}_R$ is constructed from $\boldsymbol{M}$ by keeping only its kth and $(k+n)$th rows and columns with $k \in R$.

6.1 Click cumulants, Pearson and Spearman coefficients

The comparison of click or photon-number cumulants has been used in the validation of all the GBS experiments implemented so far. This test relies on the idea that non-trivial high-order correlations of the GBS distribution are a feature that cannot be easily reproduced by classical strategies. Thus, by demonstrating that the correlations in the experimental samples match those predicted by the "ground truth" of the experiment better than any other classical model or sampler, we can build confidence in the correct functioning of the boson sampler.

Mathematically, these correlations are represented by cumulants, which are defined in terms of the joint moments of a set of random variables $(X_1, \ldots, X_M)$ as

$$\tilde{\kappa}(X_{j_1}, \ldots, X_{j_n}) = \sum_{\pi\in\mathcal{P}([n])} (-1)^{|\pi|-1}(|\pi|-1)! \prod_{b\in\pi} \left\langle \prod_{k\in b} X_{j_k} \right\rangle, \tag{S52}$$

where $\{j_1, \ldots, j_n\} \subseteq [M] = \{1, \ldots, M\}$ is a set of indices, and $n \leq M$ is the order of the cumulant. The sum runs over the collection of all partitions, π, of $[n] = \{1, \ldots, n\}$. The index b represents each block of the partition, and $|\pi|$ stands for the number of blocks in the partition. From this definition, one can readily see that the first-order cumulants are the expectation values of the random variables $\tilde{\kappa}(X_k) = \langle X_k \rangle$, while the second-order cumulants correspond to the covariances of these variables $\tilde{\kappa}(X_j, X_k) = \langle X_j X_k \rangle - \langle X_j \rangle \langle X_k \rangle$.

When estimating cumulants from a given set of samples, it is possible to use a very similar expression to Eq. (S52). We need only replace $\left\langle \prod_{k\in b} X_{j_k} \right\rangle$ by the corresponding estimator of the mean, and modify the weights $w(\pi) = (-1)^{|\pi|-1}(|\pi|-1)!$ by a factor depending on the number of samples used. We refer the reader to Ref. (*27*) for details on the definition of the unbiased estimator of the cumulants.

For the Jiuzhang experiments, the random variables are associated to the detection of a "click" (i.e. one or more photons) in the threshold detector measurement, $X_k \Leftrightarrow \hat{\mathbb{I}}_k - |0_k\rangle\langle 0_k|$. On this

account, the joint moments of these variables can be computed as

$$\left\langle \prod_{k\in b} X_{j_k} \right\rangle = \mathrm{Tr}\left[\hat{\varrho} \bigotimes_{k\in b} \left(\hat{\mathbb{I}}_{j_k} - |0_{j_k}\rangle\langle 0_{j_k}|\right)\right] = p(x_{j_k} = 1 \text{ for } k \in b). \tag{S53}$$

That is, the theoretical joint moments can be computed as marginals of the GBS distributions. It is worth noticing that Eq. (S52) is, up to a factor of $(-2)^n$ (and a shift for $n = 1$), equivalent to the expression of $\kappa(S)$ shown in Eq. (S12).

In principle, all click cumulants can be estimated if we have enough samples so that the statistical uncertainty is at least one order of magnitude lower than its ground truth value. The computation has only linear scaling with the order. However, for some distributions such as the one we face here, the cumulant values decrease exponentially with the order, making their estimation inefficient beyond a given threshold value. In addition, their number being given by the binomial $\binom{M}{d}$, increasing polynomially in the number of modes M and exponentially with the order d (until it reaches $M/2$ where their numbers start decreasing again), makes it almost impossible to collect all data or compute averages beyond a handful of orders. So far, all previous cumulant analyses of samples from experimental or classical simulation have computed full cumulants up to third order, and performed a random selection of cumulants for fourth and fifth order. In this work, after some improvement on the implementation of cumulant computation with a GPU, we were able to analyse all cumulants up to fourth order, but we are still restricted to a random sub-ensemble at fifth order, namely a subset of size $1/24$ the total number of cumulants of fifth order.

6.1.1 Scatter plots

A first tool to carry out the comparison of click cumulants is a scatter plot for each cumulant order, where one of the variables (the x-axis variable) corresponds to the ground truth cumulants, and the second variable (the y-axis variable) corresponds to the estimated cumulants from either the experimental samples or from samples generated from a classical algorithm. We show this type of plot in Figs. 2a, 3a, and 4a. If the estimated cumulants were precisely those predicted by the ground truth, all the dots in the scatter plot would lie on a straight line with unit slope and null intercept with the y-axis. On this account, estimating the parameters of a linear fit between sample and theoretical cumulants serves as a measure of how correlated a set of samples is to the ground truth of the experiments. For the validation of our sampler, we estimated the cumulants up to fifth

order using a total of 10^7 samples from both the experiments and the double-elision algorithm. We computed the corresponding linear fits (see Figs. 2a, 3a, and 4a) between theoretical and sample cumulants, and we found that our sampler generally obtains slopes closer to the ideal value than those of the experimental cumulants.

6.1.2 Pearson or Spearman

To further demonstrate that a set of estimated cumulants matches those predicted by the ground truth of the experiment, one usually shows that the Pearson or Spearman correlation coefficients between the two sets of data are close to 1 for different orders of the cumulants. This reveals the presence of a linear or monotonic relation between sample and ground truth correlations. More precisely, for each order n, let $\{\tilde{\kappa}_T^{(k)}\}_{k=1}^L$ and $\{\tilde{\kappa}_S^{(k)}\}_{k=1}^L$ be two sets of L ground truth and sample cumulants, respectively. The Pearson correlation coefficient is computed as

$$r = \frac{\sum_{k=1}^{L}(\tilde{\kappa}_S^{(k)} - \langle\tilde{\kappa}_S\rangle)(\tilde{\kappa}_T^{(k)} - \langle\tilde{\kappa}_T\rangle)}{\sqrt{\sum_{k=1}^{L}(\tilde{\kappa}_S^{(k)} - \langle\tilde{\kappa}_S\rangle)^2 \sum_{k=1}^{L}(\tilde{\kappa}_T^{(k)} - \langle\tilde{\kappa}_T\rangle)^2}}, \tag{S54}$$

where $\langle\tilde{\kappa}_S\rangle = (1/L)\sum_{k=1}^{L}\tilde{\kappa}_S^{(k)}$ and $\langle\tilde{\kappa}_T\rangle$ is similarly defined. The Spearman correlation coefficient is computed using the same expression in Eq. (S54), but replacing $\tilde{\kappa}_T^{(k)}$, $\tilde{\kappa}_S^{(k)}$ by their corresponding "rank" (i.e. by their positions when the sets $\{\tilde{\kappa}_T^{(k)}\}_{k=1}^L$ and $\{\tilde{\kappa}_S^{(k)}\}_{k=1}^L$ are ordered).

When comparing to classical models or samplers, the goal is to demonstrate that the correlation coefficients between the ground truth and classical cumulants are not as high as those between ground truth and experimental cumulants. For the validation of our sampler, we computed the Pearson correlation coefficients between sample and ground truth cumulants up to fifth order. For this computation, we used 10^7 samples from both the experiment and the double-elision algorithm (see Figs. 2b, 3b, and 4b). We find that our sampler obtains higher values of the Pearson correlation coefficient than the experimental samples for all orders.

6.2 Total click distribution

The computation and estimation of the probability of a single experimental sample $\bar{x} = (x_1, \ldots, x_M)$ with respect to the ground truth distribution of a GBS experiment are considered to be computationally hard tasks. The cost of computing these probabilities increases exponentially with the number

of clicks in the experimental samples, while their estimation requires exponentially-many of these samples. Consequently, one cannot readily validate a GBS experiment by directly estimating its corresponding probability distribution and comparing it with that predicted by the associated theoretical model. One can partially overcome this limitation by computing and estimating probabilities of "coarse-grained" detection events instead of dealing with individual, "fine-grained" experimental samples.

A coarse-grained detection event is defined by a grouping of the detection modes, which is mathematically defined by a partition $\Lambda = \{\Lambda_1, \ldots, \Lambda_\ell\}$ of $[M] = \{1, \ldots, M\}$, and a coarse grained detection pattern $\bar{C} = (C_1, \ldots, C_\ell)$. The corresponding probability is computed as

$$p(\bar{C}, \Lambda) = \sum_{\bar{x} \text{ comp. } (\bar{C},\Lambda)} p(\bar{x}), \tag{S55}$$

where the sum is taken over all detection patterns $\bar{x}$ that are "compatible" with $\bar{C}$, that is, those satisfying $\sum_{j\in\Lambda_k} x_j = C_k$ for all $k \in \{1, \ldots, \ell\}$. A particularly important type of the coarse-grained distribution is the "total click distribution", $p(C)$, obtained when the partition Λ has only one block:

$$p(C) = \sum_{|\bar{x}|=C} p(\bar{x}), \tag{S56}$$

where, let us recall, $|\bar{x}| = \sum_{k=1}^{M} x_k$ (*46*).

From Eqs. (S55) and (S56), it appears that the computation of coarse-grained probabilities is *harder* than obtaining the probability of a single detection event. However, it has been shown (*36*) that $p(\bar{C})$ can be efficiently estimated when the number of blocks, $|\Lambda|$, is not too big. In particular, the theoretical value of $p(C)$ can be efficiently estimated using phase-space techniques, and it can be experimentally estimated using polynomially-many samples. We refer the reader to Ref. (*36*) for details on the definition of the numerical phase-space methods for computing the total click distribution.

The partial validation of GBS experiments using coarse-grained detection events relies on the idea that a classical model or sampler trying to explain or simulate the experiment should be able to reproduce the coarse-grained distributions of the theoretical model of the implementation. If the experimentally estimated coarse-grained probabilities are closer to those predicted by the ground truth than those obtained by classical samplers or models, we gain more trust in the correct functioning of the boson sampler. The Jiuzhang experiments have been partially validated using

the comparison of total click distributions. This method has been able to rule out classical models such as thermal, coherent and squashed states models, and other classical samplers.

For the validation of our sampler, we estimated the double-elision total click distribution, as well as that of the experimental outcomes, using a total of 10^7 samples. The ground truth distributions were computed using a `Python` implementation of the phase-space technique for estimating the total click distribution available in the `thewalrus` library (*47*) (namely, the function `grouped_click_probabilities`). As shown in Figs. 3c, and 4c, the total click distribution of our sampler better aligns with that predicted by the ground truth.

6.3 Cross-entropy benchmarking (XEB)

A natural way of making sure that an experimental implementation of GBS is following its intended theoretical model, is to verify how similar the probability distribution of the experimental samples is to the ground truth distribution. One of the most useful statistical measures that allow one to check this similarity is the total variation distance. However, it has been shown that the accurate estimation of these quantities in the context of GBS requires a number of experimental samples that grows exponentially with the number of modes in the system, making this validation strategy inefficient. There exist other "sample-efficient" measures of similarity that act as proxies of the total variation distance without being as decisive. The XEB score is one of these measures, and has been widely used in the validation of real-world implementations of qubit-based quantum random circuit sampling (*1*).

Considering a GBS implementation using threshold detectors, let $\{\bar{x}_k\}_{k=1}^N$ be a set of experimental samples sharing the same total number of clicks, C. The XEB score for this collection of samples is defined as

$$\mathrm{XEB}(C) = \frac{1}{N}\sum_{k=1}^{N}\log\left[\binom{M}{C}\frac{p(\bar{x}_k)}{p(C)}\right], \tag{S57}$$

where $p(\bar{x})$ is the ground truth probability distribution, and $p(C)$ is the ground truth total click distribution. Note that by depending on the calculation of probabilities of individual samples, XEB is a *computationally inefficient* validation measure. This means that while it can be estimated using polynomially-many samples, one is restricted to compute it for small values of the total number of clicks, typically $C < 27$. This makes the XEB verification of bright GBS experiments (that lead to

samples with many clicks) particularly challenging.

The main idea behind the XEB validation of GBS is to compute $\mathrm{XEB}(C)$ as a function of C for a set of experimental samples, and for samples obtained using classical strategies. By showing that the experimental samples generally obtain higher XEB scores, we obtain more confidence in the possibility that the boson sampler follows the ground truth more closely than the classically generated samples. Intuitively, this is due to the fact that high XEB scores are obtained for generally high values of $p(\bar{x}_k)$, which, in principle, are obtained by samples that follow a probability distribution close to the ground truth.

It is worth mentioning that there is no evidence that obtaining a high XEB score implies that the distribution of the corresponding samples is close in total variation distance to the ground truth. For instance, there are ways of spoofing the XEB test by sampling from a reference distribution that captures some of the features of the ground truth (*48*).

For the validation of our sampler in the low-brightness regime, which corresponds to total click distributions that are supported mostly in the range $0 \leq C < 27$, we defined sets of 4000 experimental and double-elision samples for each value of C. Using these samples, we estimated the values of $\mathrm{XEB}(C)$ and plotted the results as functions of C. For the J2-P65-2 experiment in particular (see Figure 2(c)), we found that our sampler obtains XEB scores that are as high as those obtained by the experimental samples, suggesting that the distribution of the sampler is as similar to the ground truth distribution as that of the experimental samples.

REFERENCES

1. F. Arute, K. Arya, R. Babbush, D. Bacon, J. C. Bardin, R. Barends, R. Biswas, S. Boixo, F. G. S. L. Brandao, D. A. Buell, B. Burkett, Y. Chen, Z. Chen, B. Chiaro, R. Collins, W. Courtney, A. Dunsworth, E. Farhi, B. Foxen, A. Fowler, C. Gidney, M. Giustina, R. Graff, K. Guerin, S. Habegger, M. P. Harrigan, M. J. Hartmann, A. Ho, M. Hoffmann, T. Huang, T. S. Humble, S. V. Isakov, E. Jeffrey, Z. Jiang, D. Kafri, K. Kechedzhi, J. Kelly, P. V. Klimov, S. Knysh, A. Korotkov, F. Kostritsa, D. Landhuis, M. Lindmark, E. Lucero, D. Lyakh, S. Mandrà, J. R. McClean, M. McEwen, A. Megrant, X. Mi, K. Michielsen, M. Mohseni, J. Mutus, O. Naaman, M. Neeley, C. Neill, M. Y. Niu, E. Ostby, A. Petukhov, J. C. Platt, C. Quintana, E. G. Rieffel, P. Roushan, N. C. Rubin, D. Sank, K. J. Satzinger, V. Smelyanskiy, K. J. Sung, M. D. Trevithick, A. Vainsencher, B. Villalonga, T. White, Z. J. Yao, P. Yeh, A. Zalcman, H. Neven, J. M. Martinis, Quantum supremacy using a programmable superconducting processor. *Nature* **574**, 505–510 (2019).

2. D. Gao, D. Fan, C. Zha, J. Bei, G. Cai, J. Cai, S. Cao, F. Chen, J. Chen, K. Chen, X. Chen, X. Chen, Z. Chen, Z. Chen, Z. Chen, W. Chu, H. Deng, Z. Deng, P. Ding, X. Ding, Z. Ding, S. Dong, Y. Dong, B. Fan, Y. Fu, S. Gao, L. Ge, M. Gong, J. Gui, C. Guo, S. Guo, X. Guo, L. Han, T. He, L. Hong, Y. Hu, H. L. Huang, Y. H. Huo, T. Jiang, Z. Jiang, H. Jin, Y. Leng, D. Li, D. Li, F. Li, J. Li, J. Li, J. Li, J. Li, N. Li, S. Li, W. Li, Y. Li, Y. Li, F. Liang, X. Liang, N. Liao, J. Lin, W. Lin, D. Liu, H. Liu, M. Liu, X. Liu, X. Liu, Y. Liu, H. Lou, Y. Ma, L. Meng, H. Mou, K. Nan, B. Nie, M. Nie, J. Ning, L. Niu, W. Peng, H. Qian, H. Rong, T. Rong, H. Shen, Q. Shen, H. Su, F. Su, C. Sun, L. Sun, T. Sun, Y. Sun, Y. Tan, J. Tan, L. Tang, W. Tu, C. Wan, J. Wang, B. Wang, C. Wang, C. Wang, C. Wang, J. Wang, L. Wang, R. Wang, S. Wang, X. Wang, X. Wang, X. Wang, Y. Wang, Z. Wei, J. Wei, D. Wu, G. Wu, J. Wu, S. Wu, Y. Wu, S. Xie, L. Xin, Y. Xu, C. Xue, K. Yan, W. Yang, X. Yang, Y. Yang, Y. Ye, Z. Ye, C. Ying, J. Yu, Q. Yu, W. Yu, X. Zeng, S. Zhan, F. Zhang, H. Zhang, K. Zhang, P. Zhang, W. Zhang, Y. Zhang, Y. Zhang, L. Zhang, G. Zhao, P. Zhao, X. Zhao, X. Zhao, Y. Zhao, Z. Zhao, L. Zheng, F. Zhou, L. Zhou, N. Zhou, N. Zhou, S. Zhou, S. Zhou, Z. Zhou, C. Zhu, Q. Zhu, G. Zou, H. Zou, Q. Zhang, C. Y. Lu, C. Z. Peng, X. Zhu, J. W. Pan, Establishing a new benchmark in quantum computational advantage with 105-qubit Zuchongzhi 3.0 processor. *Phys. Rev. Lett.* **134**, 090601 (2025).

3. H.-S. Zhong, H. Wang, Y. H. Deng, M. C. Chen, L. C. Peng, Y. H. Luo, J. Qin, D. Wu, X. Ding, Y. Hu, P. Hu, X. Y. Yang, W. J. Zhang, H. Li, Y. Li, X. Jiang, L. Gan, G. Yang, L. You, Z. Wang, L. Li, N. L. Liu, C. Y. Lu, J. W. Pan, Quantum computational advantage using photons. *Science* **370**, 1460–1463 (2020).

4. H.-S. Zhong, Y. H. Deng, J. Qin, H. Wang, M. C. Chen, L. C. Peng, Y. H. Luo, D. Wu, S. Q. Gong, H. Su, Y. Hu, P. Hu, X. Y. Yang, W. J. Zhang, H. Li, Y. Li, X. Jiang, L. Gan, G. Yang, L. You, Z. Wang, L. Li, N. L. Liu, J. J. Renema, C. Y. Lu, J. W. Pan, Phase-programmable gaussian boson sampling using stimulated squeezed light. *Phys. Rev. Lett.* **127**, 180502 (2021).

5. L. S. Madsen, F. Laudenbach, M. F. Askarani, F. Rortais, T. Vincent, J. F. F. Bulmer, F. M. Miatto, L. Neuhaus, L. G. Helt, M. J. Collins, A. E. Lita, T. Gerrits, S. W. Nam, V. D. Vaidya, M. Menotti, I. Dhand, Z. Vernon, N. Quesada, J. Lavoie, Quantum computational advantage with a programmable photonic processor. *Nature* **606**, 75–81 (2022).

6. Y.-H. Deng, Y. C. Gu, H. L. Liu, S. Q. Gong, H. Su, Z. J. Zhang, H. Y. Tang, M. H. Jia, J. M. Xu, M. C. Chen, J. Qin, L. C. Peng, J. Yan, Y. Hu, J. Huang, H. Li, Y. Li, Y. Chen, X. Jiang, L. Gan, G. Yang, L. You, L. Li, H. S. Zhong, H. Wang, N. L. Liu, J. J. Renema, C. Y. Lu, J. W. Pan, Gaussian boson sampling with pseudo-photon-number-resolving detectors and quantum computational advantage. *Phys. Rev. Lett.* **131**, 150601 (2023).

7. H.-L. Liu, H. Su, Y. H. Deng, S. Q. Gong, Y. C. Gu, H. Y. Tang, M. H. Jia, Q. Wei, Y. K. Song, D. Z. Wang, M. Y. Zheng, F. X. Chen, L. B. Li, S. Y. Ren, X. Z. Zhu, M. H. Wang, Y. J. Chen, Y. F. Liu, L. S. Song, P. Y. Yang, J. S. Chen, H. An, L. Zhang, L. Gan, G. W. Yang, J. M. Xu, Y. M. He, H. Wang, H. S. Zhong, M. C. Chen, X. Jiang, L. Li, N. L. Liu, X. L. Su, Q. Zhang, C. Y. Lu, J. W. Pan, Gaussian boson sampling with 1,024 squeezed states in 8,176 modes. *Nature* **653**, 687–692 (2026).

8. A. W. Young, S. Geller, W. J. Eckner, N. Schine, S. Glancy, E. Knill, A. M. Kaufman, An atomic boson sampler. *Nature* **629**, 311–316 (2024).

9. R. Haghshenas, E. Chertkov, M. Mills, W. Kadow, S. H. Lin, Y. H. Chen, C. Cade, I. Niesen, T. Begušić, M. S. Rudolph, C. Cirstoiu, K. Hémery, C. Mc Keever, M. Lubasch, E. Granet, C. H. Baldwin, J. P. Bartolotta, M. Bohn, J. J. Burau, J. Cline, M. DeCross, J. M. Dreiling, C.

Foltz, D. Francois, J. P. Gaebler, C. N. Gilbreth, J. Gray, D. Gresh, A. Hall, A. Hankin, A. Hansen, N. Hewitt, C. A. Holliman, R. B. Hutson, M. Iqbal, N. Kotibhaskar, E. Lehman, D. Lucchetti, I. S. Madjarov, K. Mayer, A. R. Milne, S. A. Moses, B. Neyenhuis, G. Park, A. R. Perry, B. Ponsioen, M. Schecter, P. E. Siegfried, D. T. Stephen, B. G. Tiemann, M. D. Urmey, J. Walker, A. C. Potter, D. Hayes, G. K. L. Chan, F. Pollmann, M. Knap, H. Dreyer, M. Foss-Feig, Digital quantum magnetism on a trapped-ion quantum computer. *Nature* **653**, 56–62 (2026).

10. Y. Kim, A. Eddins, S. Anand, K. X. Wei, E. van den Berg, S. Rosenblatt, H. Nayfeh, Y. Wu, M. Zaletel, K. Temme, A. Kandala, Evidence for the utility of quantum computing before fault tolerance. *Nature* **618**, 500–505 (2023).

11. A. Bouland, B. Fefferman, C. Nirkhe, U. Vazirani, On the complexity and verification of quantum random circuit sampling. *Nat. Phys.* **15**, 159–163 (2019).

12. S. Aaronson, A. Arkhipov, "The computational complexity of linear optics," in *Proceedings of the Forty-Third Annual ACM Symposium on Theory of Computing* (Association for Computing Machinery, New York, NY, USA, 2011), pp. 333–342.

13. A. Deshpande, A. Mehta, T. Vincent, N. Quesada, M. Hinsche, M. Ioannou, L. Madsen, J. Lavoie, H. Qi, J. Eisert, D. Hangleiter, B. Fefferman, I. Dhand, Quantum computational advantage via high-dimensional Gaussian boson sampling. *Sci. Adv.* **8**, eabi7894 (2022).

14. D. Grier, D. J. Brod, J. M. Arrazola, M. B. de Andrade Alonso, N. Quesada, The complexity of bipartite gaussian boson sampling. *Quantum* **6**, 863 (2022).

15. A. Bouland, D. Brod, I. Datta, B. Fefferman, D. Grier, F. Hernández, M. Oszmaniec, Complexity-theoretic foundations of BosonSampling with a linear number of modes. *Phys. Rev. X* **16**, 021059 (2023).

16. F. Pan, P. Zhang, Simulation of quantum circuits using the big-batch tensor network method. *Phys. Rev. Lett.* **128**, 030501 (2022).

17. F. Pan, K. Chen, P. Zhang, Solving the sampling problem of the sycamore quantum circuits. *Phys. Rev. Lett.* **129**, 090502 (2022).

18. C. Oh, M. Liu, Y. Alexeev, B. Fefferman, L. Jiang, Classical algorithm for simulating experimental Gaussian boson sampling. *Nat. Phys.* **20**, 1461–1468 (2024).

19. K. Kechedzhi, S. V. Isakov, S. Mandrà, B. Villalonga, X. Mi, S. Boixo, V. Smelyanskiy, Effective quantum volume, fidelity and computational cost of noisy quantum processing experiments. *Future Gener. Comput. Syst.* **153**, 431–441 (2024).

20. M. S. Rudolph, E. Fontana, Z. Holmes, L. Cincio, Classical surrogate simulation of quantum systems with LOWESA. arXiv:2308.09109 [quant-ph] (2023).

21. T. Begušić, G. K.-L. Chan, Fast classical simulation of evidence for the utility of quantum computing before fault tolerance. arXiv:2306.16372 [quant-ph] (2023).

22. B. Villalonga, M. Y. Niu, L. Li, H. Neven, J. C. Platt, V. N. Smelyanskiy, S. Boixo, Efficient approximation of experimental Gaussian boson sampling. arXiv:2109.11525 [quant-ph] (2022).

23. J. Martínez-Cifuentes, H. de Guise, N. Quesada, Linear cross-entropy certification of quantum computational advantage in gaussian boson sampling. *PRX Quantum* **5**, 040312 (2024).

24. Z. Kolarovszki, Á. Kaposi, Z. Zimborás, M. Oszmaniec, General framework for anticoncentration and linear cross-entropy benchmarking in photonic quantum advantage experiments. arXiv:2604.15258 [quant-ph] (2026).

25. R. O'Donnell, Analysis of boolean functions. arXiv:2105.10386 [cs.DM] (2021).

26. J. Martínez-Cifuentes, K. M. Fonseca-Romero, N. Quesada, Classical models may be a better explanation of the Jiuzhang 1.0 Gaussian Boson Sampler than its targeted squeezed light model. *Quantum* **7**, 1076 (2023).

27. K. D. Smith, A tutorial on multivariate *k*-statistics and their computation. arXiv:2005.08373 [stat.ME] (2020).

28. N. Quesada, J. M. Arrazola, N. Killoran, Gaussian boson sampling using threshold detectors. *Phys. Rev. A* **98**, 062322 (2018).

29..K aposi, Z. Kolarovszki, T. Kozsik, Z. Zimborás, P. Rakyta, Polynomial speedup in Torontonian calculation by a scalable recursive algorithm. arXiv:2109.04528 [quant-ph] (2021).

30. Y. Cardin, N. Quesada, Photon-number moments and cumulants of Gaussian states. *Quantum* **8**, 1521 (2024).

31. B. Collins, S. Matsumoto, J. Novak, The Weingarten calculus. *Not. Am. Math. Soc.* **69**, 734–745 (2022).

32. J. F. Bulmer, S. Paesani, R. S. Chadwick, N. Quesada, Threshold detection statistics of bosonic states. *Phys. Rev. A* **106**, 043712 (2022).

33. P. D. Drummond, B. Opanchuk, A. Dellios, M. D. Reid, Simulating complex networks in phase space: Gaussian boson sampling. *Phys. Rev. A* **105**, 012427 (2022).

34. T. Dodd, Plot data for "A fast and frugal Gaussian Boson Sampling emulator," Zenodo (2025); https://doi.org/10.5281/zenodo.17945373.

35. T. Dodd, Sampler code for "A fast and frugal Gaussian Boson Sampling emulator," Zenodo (2025); https://doi.org/10.5281/zenodo.17945335.

36. T. Dodd, FrugalGBS, GitHub (2026); https://github.com/tomdodd4598/FrugalGBS.

37. R. A. Fisher, J. Wishart, The derivation of the pattern formulae of two-way partitions from those of simpler patterns. *Proc. Lond. Math. Soc.* **s2–33**, 195–208 (1932).

38. H. D. Ursell, The evaluation of Gibbs' phase-integral for imperfect gases. *Math. Proc. Camb. Philos. Soc.* **23**, 685–697 (1927).

39. M. Duneau, D. Iagolnitzer, B. Souillard, Decrease properties of truncated correlation functions and analyticity properties for classical lattices and continuous systems. *Commun. Math. Phys.* **31**, 191–208 (1973).

40. A. Serafini, *Quantum Continuous Variables: A Primer of Theoretical Methods* (CRC Press, 2023).

41. C. S. Hamilton, R. Kruse, L. Sansoni, S. Barkhofen, C. Silberhorn, I. Jex, Gaussian Boson sampling. *Phys. Rev. Lett.* **119**, 170501 (2017).

42. R. Kruse, C. S. Hamilton, L. Sansoni, S. Barkhofen, C. Silberhorn, I. Jex, Detailed study of Gaussian boson sampling. *Phys. Rev. A* **100**, 032326 (2019).

43. H.-S. Zhong, H. Wang, Y.-H. Deng, M.-C. Chen, L.-C. Peng, Y.-H. Luo, J. Qin, D. Wu, X. Ding, Y. Hu, P. Hu, X.-Y. Yang, W.-J. Zhang, H. Li, Y. Li, X. Jiang, L. Gan, G. Yang, L. You, Z. Wang, L. Li, N.-L. Liu, C.-Y. Lu, J.-W. Pan, Experimental raw data of "Quantum computational advantage using photons" (2020); https://quantum.ustc.edu.cn/web/en/node/915.

44. H.-S. Zhong, Y.-H. Deng, J. Qin, H. Wang, M.-C. Chen, L.-C. Peng, Y.-H. Luo, D. Wu, S.-Q. Gong, H. Su, Y. Hu, P. Hu, X.-Y. Yang, W.-J. Zhang, H. Li, Y. Li, X. Jiang, L. Gan, G. Yang, L. You, Z. Wang, L. Li, N.-L. Liu, J. J. Renema, C.-Y. Lu, J.-W. Pan, Raw data of Jiuzhang 2.0 for sharing (2021); https://quantum.ustc.edu.cn/web/en/node/951.

45. Y.-H. Deng, Y. C. Gu, H. L. Liu, S. Q. Gong, H. Su, Z. J. Zhang, H. Y. Tang, M. H. Jia, J. M. Xu, M. C. Chen, J. Qin, L. C. Peng, J. Yan, Y. Hu, J. Huang, H. Li, Y. Li, Y. Chen, X. Jiang, L. Gan, G. Yang, L. You, L. Li, H. S. Zhong, H. Wang, N. L. Liu, J. J. Renema, C. Y. Lu, J. W. Pan, Raw data of Jiuzhang 3.0 (2023); https://quantum.ustc.edu.cn/web/node/1121.

46. J. F. F. Bulmer, J. Martínez-Cifuentes, B. A. Bell, N. Quesada, Simulating lossy and partially distinguishable quantum optical circuits: Theory, algorithms, and applications to experiment validation and state preparation. *Adv. Photonics* **8**, 016010 (2026).

47. B. Gupt, J. Izaac, N. Quesada, The Walrus: A library for the calculation of hafnians, Hermite polynomials and Gaussian boson sampling. *J. Open Source Softw.* **4**, 1705 (2019).

48. C. Oh, L. Jiang, B. Fefferman, Spoofing cross-entropy measure in boson sampling. *Phys. Rev. Lett.* **131**, 010401 (2023).
